# The Constituents of *Phyllanthus emblica* Fruit Ameliorate Hyperlipidemia Through the Modulation of SREBPs, HMG‐CoA Reductase, and LDL Receptor Pathway

**DOI:** 10.1155/sci5/7941857

**Published:** 2025-12-30

**Authors:** Syed Abdul Kuddus, Md. Hasanuzzaman Shohag, Quamrul Islam Yaseen, Labiba Ahmed, Anika Tabassum Kashfia, Fatema Binte Islam, Sabrin Islam Khan, Reatul Karim, Muhammad Maqsud Hossain, Md. Ashraful Alam, Ferdous Khan

**Affiliations:** ^1^ Department of Pharmaceutical Sciences, North South University, Dhaka, Bangladesh, northsouth.edu

**Keywords:** gene expression, hyperlipidemia, *in silico*, LDL cholesterol, *Phyllanthus emblica*

## Abstract

The current study investigated the effects of dietary supplementation with antioxidant‐rich *Phyllanthus emblica* fruit powder (PEF) in high‐fat diet (HFD)–induced hyperlipidemic Wistar rats. *In silico* pharmacokinetic activity prediction and molecular docking studies were performed for several bioactive compounds of the *P. emblica* fruit. Wistar rats were arranged into four groups and fed one of the following four diets: standard diet (Control), 2% (w/w) PEF‐supplemented standard diet (Control + PEF), HFD, and 2% (w/w) PEF‐supplemented HFD (HFD + PEF). The treatment was continued for 8 weeks, after which the effects of PEF on oxidative stress, fat deposition, plasma lipids, and gene expression of relevant proteins were explored. Several proteins involved in lipid metabolism and homeostasis interact with bioactive phenolic compounds, such as ellagic acid, quercetin, catechin, kaempferol, and chrysin. The presence of these compounds in the *P. emblica* fruit was confirmed by HPLC analysis. *In vivo* experiments showed that HFD‐induced increased oxidative stress, mesenteric fat weight, and harmful plasma lipids were reduced significantly (*p* < 0.05) due to the feeding of PEF‐supplemented HFD. On the other hand, HFD‐mediated reductions in antioxidant enzyme activity and the level of HDL cholesterol were restored in rats fed with PEF. The HFD‐mediated increase in the transcript levels of SREBP‐1c, SREBP‐2, and HMGCR reductase was significantly (*p* < 0.05) suppressed by feeding PEF with a parallel increase in the expression of LDLR. However, the increased expression of LXRα, PPARγ, and FABP4 was not changed by PEF feeding, although these proteins strongly interacted with several compounds of PEF. This study demonstrated that a PEF‐supplemented diet can reduce HFD‐induced hyperlipidemia by modulating the expression of SREBP‐1c, SREBP‐2, HMG‐CoA reductase, and LDL receptor at the transcriptional level.

## 1. Introduction

Apart from genetic predisposition, excessive caloric consumption relative to energy expenditure is the primary driver of body weight gain and metabolic syndrome, which can lead to hyperlipidemia, hypertension, diabetes, and many other health issues [1]. Hyperlipidemia is characterized by increased levels of total cholesterol (TC), triacylglycerol (TG), and low‐density lipoprotein cholesterol (LDLC), along with low levels of high‐density lipoprotein cholesterol (HDLC) [2]. Increased harmful lipid levels and reduced good cholesterol levels predispose individuals to atherosclerosis, ischemic heart disease, myocardial infarction, stroke, and related cardiovascular disorders [3, 4]. Currently, for the management of hyperlipidemia, synthetic molecules such as statins, fibrates, niacin, and PCSK9 inhibitors are the most commonly used drugs [5]. However, their adverse effects, especially those associated with long‐term use, highlight the need for safer alternatives that can reduce hyperlipidemia and restore lipid homeostasis, with greater safety and efficacy.

Phytochemicals from different medicinal plants are generally safer and less toxic than most synthetic drugs and have gained increased researchers’ attention [6]. Among these, *Phyllanthus emblica* fruit, also known as Indian gooseberry (Family: *Euphorbiaceae*), has shown notable therapeutic potential, predominantly due to its high antioxidant content [7, 8]. In addition to primary metabolites, the fruit contains a diverse array of bioactive secondary metabolites, including vitamins, tannins, and phenolic and nonphenolic compounds [9]. Through phytochemical screening, researchers demonstrated the presence of phenolic compounds, including quercetin, gallic acid, kaempferol, ellagic acid, myricitrin, myricetin, catechin, and chrysin, as well as nonphenolic compounds, such as ascorbic acid, caffeic acid, betaine, trigonelline, betulinic acid, and oleanolic acid [10–12]. Moreover, using HPLC‐DAD analysis, researchers have estimated the amount of gallic acid (65.12 mg/100 g dry extract), catechin hydrate (55.03 mg/100 g dry extract), caffeic acid (9.77 mg/100 g dry extract), and ellagic acid (74.83 mg/100 g dry extract) in the alcoholic extract of amla fruit [13]. Preclinical and clinical investigations have revealed that quercetin, kaempferol, ellagic acid, catechin, and myricetin mitigate hyperlipidemia and hyperglycemia through their antioxidant and anti‐inflammatory properties. For example, quercetin ameliorates chronic disease‐induced inflammatory and oxidative damage of several organs [14]. A recent study showed that kaempferol and quercetin outperformed metformin in controlling blood glucose and suppressing oxidative stress in diabetic mice [15]. In mice, an ellagic acid–supplemented diet stimulated antioxidant defense and hepatic fat metabolism, which eventually restored blood lipids through the modulation of crucial proteins and transcription factors [16]. Another study revealed that catechin, by reducing oxidative stress, protects vascular endothelial cells from ox‐LDL‐induced atherosclerotic plaque development in ApoE™/transgenic mice by activating the Nrf2/SLC7A11/GPX4 pathway [17]. *Tinospora sinensis* extract containing caffeic acid, berberine, myricetin, and ferulic acid effectively ameliorated streptozotocin‐induced diabetes in rats by lowering oxidative stress, blood glucose, and inflammation as well as restoring the function of pancreatic β‐cells [18].

Clinical studies have demonstrated that dietary supplementation with *P. emblica* markedly ameliorates endothelial cell dysfunction, reduces inflammation, oxidative stress, and lipid peroxidation, and decreases plasma lipid levels in human volunteers [19–21]. Similarly, a number of murine models of kidney and liver diseases reported that *P. emblica* reduces oxidative stress along with an increase of antioxidant enzyme activity [22–25]. Additionally, through cell culture studies, researchers showed that *P. emblica* and its bioactive constituents effectively prevented the increase of reactive oxygen species (ROS) through restoring the glutathione levels and the activities of several enzymes, including superoxide dismutase (SOD), catalase, and glutathione peroxidase (GPx) [26–28]. Despite these promising results, the molecular mechanisms through which *P. emblica* and its bioactive phytochemicals modulate lipid metabolism and homeostasis remain poorly understood.

The physiology of lipid homeostasis and the pathogenesis of hyperlipidemia involve a complex network of transcription factors, receptors, and enzymes [29, 30]. Therefore, the role of the abovementioned bioactive phenolics in modulating those networks is crucial in understanding the molecular mechanisms of *P. emblica*. The emergence of computational biology and molecular docking has facilitated the use of valuable resources for exploring and predicting protein–ligand interactions and therapeutic targets [31, 32]. Recent *in silico* computational studies have identified interactions between certain compounds of *P. emblica* and some proteins having roles in lipid homeostasis and metabolic syndrome [33, 34]. However, the predicted pharmacological benefits should be validated through *in vitro* and *vivo* studies to fully comprehend the molecular mechanisms at play in the body of living organisms. This study, through the integration of *in silico*, *in vitro*, and *in vivo* methodologies, aims to investigate the effects of *P. emblica* phytochemicals on hyperlipidemia by exploring their interaction with several proteins that have implications in diet‐induced hyperlipidemia.

## 2. Materials and Methods

### 2.1. Chemicals and Reagents

Gallic acid, ellagic acid, catechin, kaempferol, chrysin, quercetin, myricitrin, betaine, and other standard compounds used in the HPLC study were purchased from Sigma‐Aldrich (MO, USA). HPLC‐grade solvents like acetonitrile, methanol, and ethanol were purchased from Merck (Darmstadt, Germany). Folin–Ciocalteu reagent, thiobarbituric acid, trichloroacetic acid, sulfanilamide, and malondialdehyde (MDA) tetrabutylammonium were obtained from Wako Chemicals (Osaka, Japan). All the commercial reagents for the assay of plasma lipids including TC, HDLC, LDLC, and triglyceride were collected from Plasmatec, UK. Reagents for RNA isolation, purification, conversion to cDNA, and SYBR Green Master Mix for performing quantitative real‐time PCR (qPCR) were obtained from ThermoFisher Scientific, USA. Oligonucleotides, used as primers, were bought from Macrogen Inc. (Seoul, Korea). Additional reagents and chemicals required for oxidative stress measurement and antioxidant enzyme activity assay were procured from Scharlau, Spain.

### 2.2. Selection of Phytochemicals and *In Silico* Pharmacokinetic Evaluation

Through a literature survey, 21 phytochemicals of *Phyllanthus emblica* fruit were selected for *in silico* study based on their antioxidant, anti‐inflammatory, antihyperlipidemic, and antihyperglycemic properties and overall roles in cardiovascular health. The pharmacological properties of 21 selected compounds, including gallic acid, ellagic acid, quercetin, and betaine, with the reported protein targets are presented in Supporting Table 1 (S1). The official SMILES codes for these compounds were obtained from PubChem (https://pubchem.ncbi.nlm.nih.gov). The *in silico* pharmacokinetic properties of these molecules were predicted using SwissADME (https://www.swissadme.ch). This online tool uses “Lipinski’s Rule of Five” for predicting drug‐likeness, which refers to the prospect of a drug candidate, for oral administration, based on its molecular weight, solubility, and permeability [35]. The absorption pattern from the gastrointestinal tract and the probability of penetration into the brain through the blood–brain barrier (BBB) were predicted using the Egan BOILED‐Egg model. For the construction of the Egan BOILED‐Egg model, the Wildman and Crippen partition coefficient (WLOGP) value of a specific compound was plotted as a function of its topological polar surface area (TPSA) [36]. Atorvastatin, a lipid‐lowering statin, was used as the standard for comparison.

### 2.3. Molecular Docking Analysis of Selected Phytochemicals

Compounds with acceptable pharmacokinetic properties and drug‐likeness were subjected to molecular docking studies with eight proteins with vital roles in lipid metabolism and homeostasis. These proteins include sterol regulatory element‐binding protein 1c (SREBP‐1c), sterol regulatory element‐binding protein 2 (SREBP‐2), retinoid X receptor alpha (RXRα), liver X receptor alpha (LXRα), peroxisome proliferator–activated receptor gamma (PPARγ), HMG‐CoA reductase (HMGCR), low‐density lipoprotein receptor (LDLR), and fatty acid‐binding protein 4 (FABP4). For molecular docking analysis, the 3D structures of these proteins were downloaded in PDB format from the UniProt (https://www.uniprot.org) and RCSB (https://www.rcsb.org) websites. The structures of the ligands (compounds of *P. emblica* fruit) were downloaded in SDF format from PubChem online (https://pubchem.ncbi.nlm.nih.gov) and converted to PDB format for further processing. Proteins and ligands were processed for docking analysis using AutoDockTools 1.5.7 [37]. Subsequently, blind docking between proteins and ligands was performed to calculate the docking scores (kcal/mol) using the CB‐Dock2 server, and 2D images of the interaction were generated using BIOVIA Discovery Studio (Version 21) [38].

### 2.4. Preparation of the Standard Diet, High‐Fat Diet (HFD), and *P. emblica* Fruit Powder (PEF) Supplemented Diet

*Phyllanthus emblica* fruit, which was purchased from a local market in Bangladesh, was identified (ID number: BD‐NH2022PE06) by a professional plant taxonomist at the National Herbarium, Dhaka, Bangladesh. After cutting the fruits into small pieces, they were air‐dried (40°C) and pulverized into fine powder. The composition of the AIN‐76A diet formulated by the American Institute of Nutrition was used as a framework for preparing a normal diet (Control) and HFD [39]. 2.0% (w/w) PEF‐supplemented normal diet (Control + PEF) and 2.0% (w/w) PEF‐supplemented HFD (HFD + PEF) were prepared by mixing 20 g of *P. emblica* dried fruit powder with 980 g of normal diet and 980 g of HFD, respectively. The quantity of ingredients was maintained to ensure that each kg of a normal diet (Control) and a normal diet supplemented with 2% PEF (Control + PEF) contained 3782 kcal, with fat providing 11.9% [40]. Similarly, each kg of HFD and HFD supplemented with 2% PEF (HFD + PEF) contained 5192 kcal, in which fat contributed 52.4%. The compositions of all 4 types of diets with energy content are mentioned in Supporting Table 2 (S2).

### 2.5. Measurement of Total Phenolic Content (TPC), Total Flavonoid Content (TFC), Radical Scavenging Activity, and Ferric Reducing Antioxidant Power (FRAP)

To prepare an ethanolic extract suitable for *in vitro* analysis, the fruit powder of *P. emblica* was soaked in ethanol. Then, ethanol was evaporated using a rotary evaporator to prepare a crude extract, which was used for the assay of TPC, TFC, DPPH, and ABTS radical scavenging activities, and FRAP. The TPC of the extract was determined according to a previous report using Folin–Ciocalteu reagent in the spectrophotometric method [41]. The TFC was measured by aluminum chloride colorimetric assay, described by Phuyal et al. [42]. The DPPH radical scavenging activity of *P. emblica* fruit extract was quantified using a previously described method. Briefly, in this method, several solutions with different concentrations of *P. emblica* fruit extract were allowed to react with a 40 μg/mL solution of DPPH for 30 min under light‐protected conditions [43]. To evaluate the ABTS radical scavenging activity, 2,2′‐azino‐bis (3‐ethylbenzothiazoline‐6‐sulfonic acid) (ABTS^+^) ions were allowed to react with 10 μL extract for 15 min under light‐protected conditions, and the radical scavenging potency was estimated based on the extract’s ability to decolorize the ABTS reagent according to a previous report [44]. A reagent containing 300 mM acetate, 10 mM 2,4,6‐tripyridyl‐s‐triazine (TPTZ), and 40 mM FeCl_3_ at a ratio of 10 : 1 : 1 was prepared for the FRAP assay. Then, 900 μL of this reagent was mixed with 30 μL solution of *P. emblica* fruit extract, and the final volume was made 1000 μL with the addition of water. The absorbance changes of the extract were then determined according to a previous method [45]. In the DPPH and ABTS radical scavenging assays as well as the FRAP assay, ascorbic acid and butylated hydroxytoluene (BHT) were used as standard compounds.

### 2.6. HPLC Analysis

To detect the presence of several polyphenolic compounds in *P. emblica* fruit extract, reversed‐phase HPLC (LC‐20A, Shimadzu, Japan) was employed according to the method of Ahmed et al. [6] with a few modifications [6]. Briefly, for chromatographic separation, the C18 column (Luna Phenomenex) was maintained at 34°C in an HPLC column oven (CTO‐20A). A binary solvent pump (LC‐20AT), photodiode array detector (SPD‐M20A), and automatic injection unit (SIL‐20A HT) were equipped with the HPLC system. HPLC‐grade acetonitrile containing 1% acetic acid (solvent A) and Milli‐*Q* water containing 1% acetic acid (solvent B) was used as the mobile phase. The solvents were vacuum‐filtered through a 0.45‐μm nylon membrane filter and degassed for 15 min. The flow rate of the mixture of mobile phases was set at 0.5 mL/min, and a volume of 20 μL was injected for analysis. Gradient elution of the *P. emblica* fruit extract solution (10 mg/mL) and standard stock solution in methanol (10–50 μg/mL) was performed separately for 50 min, where the relative proportion of solvent B was gradually increased. LC solution software was used to acquire and analyze the chromatograms of standard and unknown compounds [11].

### 2.7. Study Design and Animal Feeding

Male Wistar rats (8–9 weeks old and 190 ± 8 g body weight) were reared in separate cages placed in a room maintained between 22°C and 25°C and a 12 h light/dark cycle with an adequate supply of food and water. The comfort and well‐being of the rats were ensured by a protocol approved by the IACUC of North South University, Bangladesh (ACE‐029‐2021). The rats were arbitrarily arranged into 4 groups; each group consisted of 7 animals.

 Control: Consumed standard diet for rats
 Control + PEF: Consumed standard diet supplemented with 2% (w/w) PEF
 HFD: Consumed HFD
 HFD + PEF: Consumed HFD supplemented with 2% (w/w) PEF

The feeding was continued for 8 weeks. Body weight change and food and water intake were monitored daily throughout the treatment period [46].

### 2.8. Measurement of Blood Glucose, Collection of Serum, Liver, and Adipose Tissues

At the end of the 8‐week treatment period, the fasting blood sugar (FBS) level was checked using a Bionime GM700S glucometer (Berneck, Switzerland). The rats were then euthanized by ketamine injection (100 mg/kg, SC) and sacrificed for blood and other sample collection. The serum was separated by centrifugation (at 8000 × *g*) of the blood for 15 min and stored at −18°C for later investigations. The fatty tissue attached to the surface of the liver was separated, and the wet weight was recorded. A portion of hepatic tissue from each rat was also collected for the extraction of RNA, and another portion was stored in 10% neutral buffered formalin (NBF) for liver histology. The peritoneal, epididymal, and mesenteric adipose tissues were collected cautiously and rinsed with cooled PBS, and then, the weights were taken according to a previous report [47].

### 2.9. Oxidative Stress and Plasma Lipid Assessment

The concentration of MDA was assayed spectrophotometrically by detecting a complex between MDA and thiobarbituric acid [48]. Griess reagent, which consists of an acidic solution of 1.5% (w/v) sulfanilamide and 1% (w/v) N‐(1) naphthyl ethylenediamine (NED), was used to measure the plasma level of nitric oxide. The absorbance of the pink complex (*λ*max = 548 nm) was measured, and the plasma NO level was computed using a standard curve [49]. Advanced oxidation protein product (AOPP) concentration was measured at 340 nm according to a previous method [50]. Ellman’s reagent was used to assay the concentration of reduced glutathione (GSH) in the samples, which measures the absorbance of a yellow anion of 2‐nitro‐5‐thiobenzoic acid (TNB) at 410 nm [51]. SOD activity was recorded at 560 nm by following the procedure developed by Kakkar et al. with some modifications [52]. The activity of catalase was measured using a commercial kit by Abcam (Cambridge, UK), which measures the concentration of remnant hydrogen peroxide as a function of enzymatic activity. In this method, unreacted H_2_O_2_ reacts with a probe to produce a complex that can be measured at 570 nm [53]. The concentrations of TC, TG, LDLC, and HDLC were assayed in plasma samples using the diagnostic kits of Plasmatec Laboratories (Allington, UK).

### 2.10. Quantification of Gene Expression

An RNA extraction kit of ThermoFisher Scientific (USA) was used to isolate mRNA from hepatic tissue. After measuring the concentration of mRNA using NanoDrop2000 (Bio‐Rad, USA), 1 μg mRNA from each rat was utilized as a template to generate complementary DNA using a cDNA Synthesis Kit (ThermoFisher Scientific) in a thermal cycler. The cDNA was then used for qPCR using a qPCR Master Mix purchased from Thermo Scientific (USA). To quantify the transcript levels of target genes, forward and backward primers were designed using the Primer3Plus online tool and are listed in Table 1. Real‐time PCR was performed using the program developed by Khan et al. [54] using the PCR system of Bio‐Rad Laboratories Inc. (California, USA). The data were collected and analyzed, and transcript levels were calculated using CFX ManagerTM, which was developed by the same manufacturer. The mRNA levels of the target protein were assessed by normalizing to the mRNA level of β‐actin for the same sample.

**Table 1:** The sequences of forward and reverse primers used in this study for quantitative real‐time PCR.

Gene (GenBank accession no.)	Forward	Reverse
SREBP‐1c (NM_001276707.1)	5′‐GGC​ATG​AAA​CCT​GAA​GTG​GT‐3′	5′‐TGC​AGG​TCA​GAC​ACA​GGA​AG‐3′
SREBP‐2 (NM_001033694.2)	5′‐TCA​TTC​AGC​CAG​GTC​CCA​TT‐3′	5′‐CTG​AAG​GTC​GGG​GTG​ATC​AT‐3′
RXRα (NM_012805.3)	5′‐ACA​CCC​ATC​GAC​ACT​TTC​CT‐3′	5′‐ATG​ATG​GCG​AGG​ATG​GTG​AT‐3′
LXRα (NM_012805.3)	5′‐TCG​ACA​AGA​GAC​AGC​GGA​AC‐3′	5′‐GTC​AAG​CAG​CAG​ACA​AGC​AG‐3′
PPARγ (AB019561.1)	5′‐CCC​TGG​CAA​AGC​ATT​TGT​AT‐3′	5′‐GAA​ACT​GGC​ACC​CTT​GAA​AA‐3′
HMGCR (NM_013134.2)	5′‐CAT​GCT​GCC​AAC​ATC​GTC​A‐3′	5′‐TTG​TGG​GAC​TTG​CTT​CAT‐3′
LDLR (NM_175762.3)	5′‐CAG​CTG​CTG​TGT​CAC​TGA​AG‐3′	5′‐CTT​GGA​CTT​GGG​AGG​ACA​CT‐3′
FABP4 (NM_053365.1)	5′‐CCT​TTG​TGG​GGA​CCT​GGA​AA‐3′	5′‐TGA​CCG​GAT​GAC​GAC​CAA​GT‐3′
β‐actin (V01217.1)	5′‐AGC​CAT​GTA​CGT​AGC​CAT​CC‐3′	5′‐CTC​TCA​GCT​GTG​GTG​GTG​AA‐3′

### 2.11. Histology

For the histopathological evaluation of the effect of *P. emblica* on HFD‐induced adipogenesis, the hepatic tissues of rats from each group were fixed in 10% NBF, followed by ethanol and xylene treatment. The tissues were carefully embedded in paraffin slabs. The tissues were then cut with a microtome into thin (5 μm) transparent slices, which were subsequently attached to glass slides and then stained with hematoxylin/eosin (H&E). All slides with stained tissues were photographed and analyzed carefully under a light microscope at 40× magnification (ZEISS Axioscope) [55]. Additionally, the percentage of area covered by lipids in a specific photograph was measured using ImageJ software for quantitative analysis.

### 2.12. Statistical Analysis

The *in vitro* analysis of *P. emblica* powder (PEF) extract was done in triplicate (*n* = 3). *In vivo* tests including body weight change, biochemical assay, and the quantification of mRNA levels were carried out in sextuplicate (*n* = 6). The results are shown as mean ± standard error of the mean (SEM). For the detection of significant variation between 2 groups, data were analyzed by one‐way ANOVA using GraphPad Prism.

## 3. Results

### 3.1. Evaluation of Pharmacokinetic and Medicinal Properties of Selected Compounds

**Figure 1:**
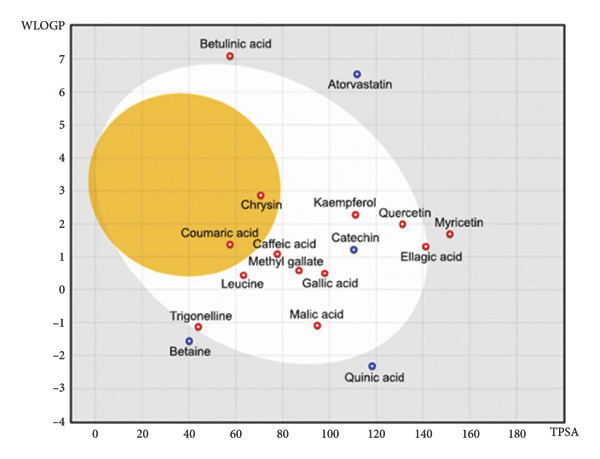
Egan’s BOILED‐egg model of selected phytochemicals of *P. emblica* fruit. The model was constructed by plotting the octanol/water partition coefficient value (WLOGP) as a function of topological polar surface area (TPSA). The white area represents a high possibility of passive diffusion through the gastrointestinal tract, and the yellow area indicates a high chance of brain penetration. The compounds indicated by the blue circle are substrate and red circle are nonsubstrate of p‐glycoprotein (PGP), an ATP‐binding cassette transporter protein, involved in the movement of molecules across the cell membrane.

The molecular weight, number of hydrogen bond acceptors (HBA), number of hydrogen bond donors (HBD), octanol/water partition coefficient value (WLOGP), TPSA, number of Lipinski’s violations (LVs), solubility, and bioavailability score (BS) of 21 compounds obtained from the SWISSADME analysis are listed in Table 2. Additional properties such as the ability to inhibit several cytochrome enzymes, lead‐likeness, and synthetic accessibility are presented in Supporting Table 3 (S3). Among the 21 selected compounds, 16 were found to be bioavailable after oral administration, as suggested by the BSs (Table 2) and the Eagan BOILED‐Egg model (Figure 1). The bioavailable compounds include gallic acid, ellagic acid, betaine, quercetin, trigonelline, myricetin, leucine, kaempferol, quinic acid, catechin, caffeic acid, chrysin, malic acid, coumaric acid, methyl gallate, and betulinic acid. Moreover, chrysin and coumaric acid could enter the brain through the BBB (Figure 1). However, five compounds: myricitrin, paromomycin, rutin, ellagitannin, and pedunculagin were not found in the “BOILED‐Egg” due to their poor gastrointestinal absorption characteristics and high TPSA values (> 150) (Table 2). These 5 compounds also have greater violation (≥ 2) of Lipinski’s rule of five and low BSs (Table 2). Therefore, these compounds were not considered for further *in silico* analysis. Hence, molecular docking studies were conducted on these 16 compounds.

**Table 2:** Properties of selected compounds obtained from the SwissTargetPrediction tool.

No.	Molecule name	Molecular weight	Solubility	HBA	HBD	WLOGP	TPSA/Å^2^	LV	BS
1	Gallic acid	170.1	High	5	4	0.50	97.9	0	0.56
2	Ellagic acid	302.2	Moderate	8	4	1.24	140.3	0	0.55
3	Quercetin	302.2	Moderate	7	5	2.01	131.3	0	0.55
4	Betaine	117.2	High	2	0	−1.55	40.1	0	0.55
5	Trigonelline	137.1	High	2	0	−0.33	44.0	0	0.55
6	Myricitrin	464.4	Moderate	12	8	0.02	210.5	2	0.17
7	Myricetin	318.2	Moderate	8	6	1.71	151.6	1	0.55
8	Leucine	131.2	High	3	2	0.44	63.3	0	0.55
9	Kaempferol	286.2	Moderate	6	4	2.30	111.1	0	0.55
10	Paromomycin	615.6	High	19	13	−8.86	347.3	3	0.17
11	Rutin	610.5	High	16	10	−1.87	269.4	3	0.17
12	Caffeic acid	180.2	High	4	3	1.19	77.8	0	0.56
13	Quinic acid	192.2	High	6	5	−2.32	118.2	0	0.56
14	Catechin	290.3	High	6	5	1.54	110.4	0	0.55
15	Chrysin	254.2	Moderate	4	2	2.71	70.7	0	0.55
16	Methyl gallate	184.2	High	5	3	0.59	86.9	0	0.55
17	Ellagitannin	992.7	Low	27	13	−2.82	447.1	3	0.17
18	Malic acid	134.1	High	5	3	−1.09	94.8	0	0.56
19	Pedunculagin	784.5	Moderate	22	13	−0.45	377.4	3	0.17
20	Coumaric acid	164.2	Moderate	3	2	1.49	57.2	0	0.55
21	Betulinic acid	456.7	Low	3	2	7.08	57.5	1	0.17
22	Atorvastatin	558.6	Low	6	4	4.24	111.8	1	0.56

Abbreviations: BS = bioavailability score, HBA = hydrogen bond acceptor, HBD = hydrogen bond donor, LV = Lipinski’s violation number, TPSA = topological polar surface area, WLOGP = value of octanol/water partition coefficient.

### 3.2. Interactions of Selected Molecules With Target Proteins

**Figure 2:**
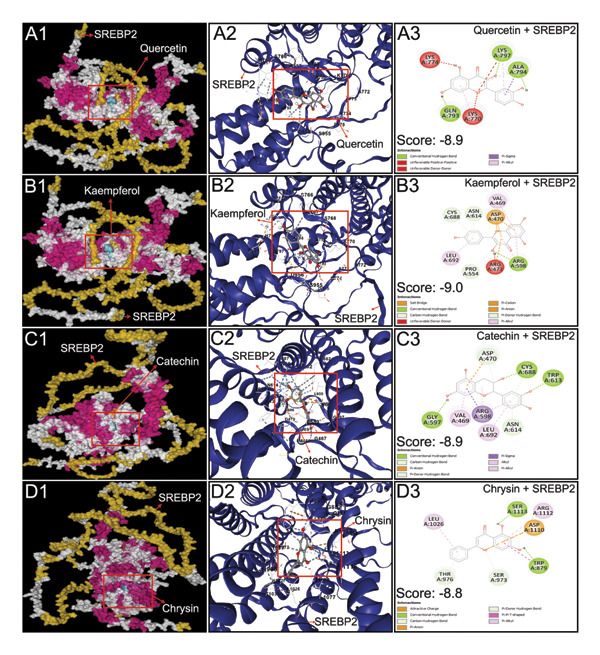
Interactions of quercetin, kaempferol, catechin, and chrysin with sterol regulatory element‐binding protein 2 (SREBP‐2). Output obtained from top 5 docking poses between SREBP‐2 and corresponding ligand using CB Dock 2 server. The ligand‐binding pose demonstrated the highest affinity of binding with the least root mean square deviation (RMSD) with a value of ≤ 2 Å. The protein–ligand interaction in 3D structures was visualized in Discovery Studio, Version 21 (BIOVIA, Germany). The left panel indicates the surface model, the middle panel cartoon model, and the right panel 2D models of the protein–ligand interaction. In this figure, A1, A2, and A3 demonstrate the interaction of SREBP2 with quercetin; B1, B2, and B3 with kaempferol; C1, C2, and C3 with catechin; and D1, D2, and D3 with chrysin.

**Figure 3:**
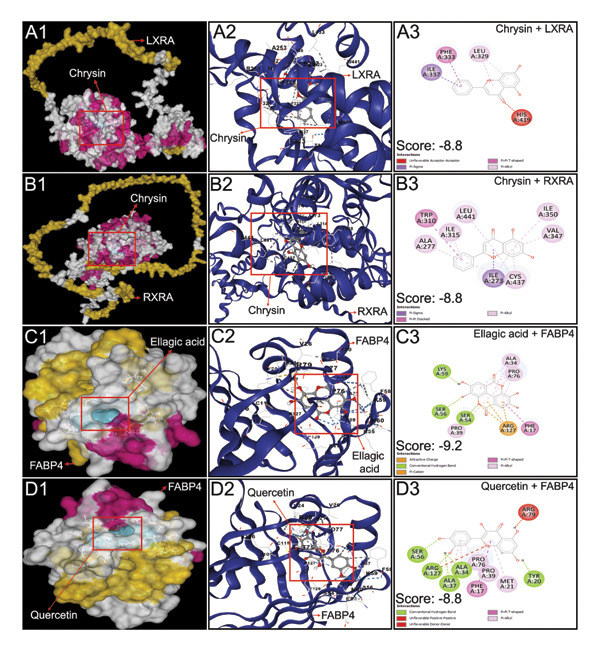
Interactions of LXRα with chrysin, RXRα with chrysin, FABP4 with ellagic acid, and FABP4 with quercetin. Output obtained from top 5 docking poses between protein and corresponding ligand using CB Dock 2 server. The ligand‐binding pose demonstrated the highest affinity of binding with the least root mean square deviation (RMSD) with a value of ≤ 2 Å. The protein–ligand interaction in 3D structures was visualized in Discovery Studio, Version 21 (BIOVIA, Germany). The left panel indicates the surface model, the middle panel cartoon model, and the right panel 2D models of the protein–ligand interaction. In this figure, A1, A2, and A3 demonstrate the interaction between LXRα and chrysin; B1, B2, and B3 between RXRα and chrysin; C1, C2, and C3 between FABP4 and ellagic acid; and D1, D2, and D3 between FABP4 and quercetin.

All 16 compounds from *P. emblica* with acceptable pharmacokinetic properties and drug‐likeness were subjected to molecular docking studies with eight proteins, as described in the materials and methods section. Atorvastatin, a lipid‐lowering statin, was used as a standard for comparison. Quercetin, kaempferol, catechin, and chrysin strongly interacted with SREBP‐2 (Table 3 and Figure 2). Chrysin also interacted with RXRα and LXRα strongly (Table 3 and Figure 3). Ellagic acid and quercetin strongly interacted with FABP4 (Table 3 and Figure 3). The docking scores of protein ligand interaction, obtained from “AutoDock Vina,” are considered strong when they are lower than −8.0 kcal/mol [56]. The docking scores (AKA binding energy or binding score) of 5 bioactive molecules that interacted strongly with the abovementioned proteins are listed as minimum binding energies, expressed as kcal/mol, and are presented in Table 3. All four flavonoids (catechin, chrysin, kaempferol, and quercetin) found in HPLC analysis of PEF strongly interacted with SREBP‐2 (Figure 2). Both LXRα and RXRα strongly interacted with chrysin; however, FABP4 strongly interacted with both ellagic acid and quercetin (Figure 3).

**Table 3:** Docking scores (kcal/mol), calculated by AutoDock Vina, between the target proteins and selected compounds of *P. emblica* fruit.

Sl no.	Name	SREBP1c (kcal/mol)	SREBP2 (kcal/mol)	RXRα (kcal/mol)	LXRα (kcal/mol)	PPARγ (kcal/mol)	HMGCR (kcal/mol)	LDLR (kcal/mol)	FABP4 (kcal/mol)
1	Gallic acid	−5.3	−6.7	−5.3	−5.6	−5.6	−6.0	−5.3	−5.7
2	Ellagic acid	−7.7	−8.4	−7.0	−8.2	−8.6	−6.7	−7.5	−9.2
3	Quercetin	−7.9	−8.9	−7.5	−8.1	−8.0	−7.7	−7.2	−8.8
4	Betaine	−3.6	−4.5	−3.6	−4.0	−3.7	−3.9	−2.9	−3.6
5	Trigonelline	−5.2	−5.5	−5.1	−5.1	−4.8	−5.1	−4.2	−5.1
6	Myricetin	−4.5	−5.0	−4.4	−4.8	−4.6	−4.8	−3.8	−4.7
7	Leucine	−7.4	−7.4	−6.3	−7.6	−8.0	−6.4	−6.1	−5.1
8	Kaempferol	−7.4	−9.0	−7.4	−7.9	−8.1	−7.4	−7.1	−8.1
9	Caffeic acid	−5.9	−6.2	−5.6	−5.9	−5.8	−5.9	−4.5	−5.8
10	Quinic acid	−5.5	−6.7	−5.5	−5.6	−5.5	−6.0	−5.1	−6.0
11	Catechin	−7.6	−8.9	−8.0	−7.9	−7.9	−7.3	−6.6	−8.3
12	Chrysin	−7.7	−8.8	−8.8	−8.8	−7.8	−8.0	−7.1	−8.1
13	Methyl gallate	−4.3	−4.6	−5.2	−4.3	−4.6	−4.6	−3.5	−3.9
14	Malic acid	−4.7	−4.7	−5.2	−5.0	−4.3	−4.5	−3.6	−4.4
15	Coumaric acid	−5.4	−6.7	−4.9	−5.2	−5.7	−5.2	−4.4	−5.9
16	Betulinic acid	−6.0	−6.2	−6.4	−5.9	−5.7	−5.5	−4.8	−5.9
17	Atorvastatin	−8.3	−8.0	−7.0	−6.8	−9.0	−7.1	−6.8	−7.1

*Note:* The docking scores for myricitrin, paromomycin, rutin, ellagitannin, and pedunculagin were not calculated due to their poor bioavailability scores and higher violations of Lipinski’s (LV) rule. The docking scores of atorvastatin were also calculated for comparison.

Abbreviations: FABP4 = fatty acid‐binding protein 4, HMGCR = HMG‐CoA reductase, LDLR = low‐density lipoprotein receptor, LXRα = liver X receptor alpha, PPARγ = peroxisome proliferator–activated receptor gamma, RXRα = retinoid X receptor alpha, SREBP‐1c = sterol regulatory element‐binding protein 1c, SREBP‐2 = sterol regulatory element‐binding protein 2.

### 3.3. TPC and TFC and Antioxidant Activity Assay

The TPC and TFC of PEF were found to be 213.3 ± 8.2 mg GAE/g (gallic acid equivalent per gram extract) and 57.4 ± 5.7 mg QE/g (quercetin equivalent per gram extract). These values indicate that the extract prepared from the dried powder of *P. emblica* fruit is a rich source of phenolic compounds along with a substantial amount of flavonoids, due to which PEF exhibited strong antioxidant activity. The DPPH radical scavenging activity of PEF is slightly higher than that of BHT, although significantly (*p* < 0.05) lower than that of ascorbic acid. However, PEF exhibited significantly (*p* < 0.05) higher ABTS radical scavenging and ferric‐reducing antioxidant activities than BHT. On the other hand, ascorbic acid outperformed PEF in all of the aforementioned activities (Table 4).

**Table 4:** Total phenolic content, radical scavenging activity, and reducing properties of the extract of *Phyllanthus emblica* fruit powder (PEF).

	Total phenolic content (mg GAE/g)	Total flavonoid content (mg QE/g)	DPPH radical scavenging activity IC_50_ (μg/mL)	ABTS radical scavenging activity IC_50_ (μg/mL)	FRAP assay (mg AAE/g)
PEF	213.3 ± 8.2	57.4 ± 5.7	2.81 ± 0.26^a^	2.48 ± 0.12^a^	492.0 ± 8.5^a^
BHT	NA	NA	2.63 ± 0.29^a^	1.85 ± 0.11^b^	449.2 ± 12.3^b^
Ascorbic acid	NA	NA	3.82 ± 0.094^b^	3.68 ± 0.29^c^	992.5 ± 31.2^c^

*Note:* Values are shown as the average of three experiments. For statistical comparison, data were evaluated in “one‐way ANOVA” that was followed by the Newman–Keuls post hoc test. Values having significant (*p* < 0.05) differences within the same column are represented by different superscript letters.

### 3.4. HPLC Detection of Polyphenolic Phytochemicals

**Figure 4:**
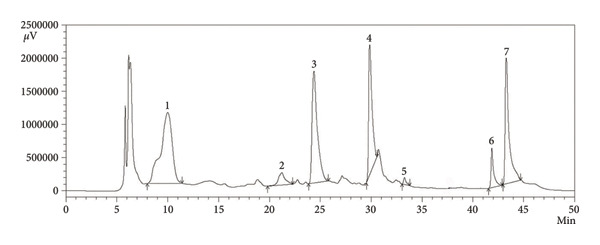
HPLC chromatogram of ethanolic extract of the dried powder of *P. emblica* fruit. Peaks: 1, gallic acid; 2, leucine; 3, catechin; 4, ellagic acid; 5, chrysin; 6, kaempferol; 7, quercetin.

By considering the bioavailability characteristics, drug‐likeness, and strong interaction with target proteins, as observed in the *in silico* study, we attempted to detect the presence of several compounds using HPLC. In the comparison of the retention time from the chromatograms of the extract prepared by *P. emblica* dried fruit powder with the chromatogram of standard stock solutions, we detected the presence of seven compounds, including one phenolic acid (gallic acid), one amino acid (leucine), one polyphenol (ellagic acid), and four flavonoids (catechin, chrysin, kaempferol, and quercetin) (Figure 4). The chromatogram of the 16 standard compounds is included in the Supporting Figure (available here). The amount of gallic acid, catechin, ellagic acid, and quercetin in the extract of *P. emblica* fruit was relatively higher than that of leucine, kaempferol, and chrysin (Table 5). The chromatographic detection of compounds was repeated 3 times, and one of the representative chromatograms is shown in Figure 4.

**Table 5:** Contents of bioactive phytochemicals in the alcoholic extract of *Phyllanthus emblica* dried fruit powder (*n* = 3).

Compound name	Alcoholic extract of *P. emblica*	
	Amount (mg/100 g of dried extract)	Relative standard deviation (%)
Gallic acid	73.21	1.53
Leucine	11.37	0.062
Catechin	45.22	1.05
Ellagic acid	61.54	1.74
Chrysin	5.12	0.085
Kaempferol	18.22	0.075
Quercetin	78.23	2.13

### 3.5. Effect of PEF on the Consumption of Food, Body Weight, and Fat Weight

The changes in body weight, liver wet weight, and fat weight due to changes in feeding are presented in Table 6. HFD‐induced gains in body weight, liver weight, and adipose tissue weight were not associated with the quantity of food but rather with the calorie content of the food, which was related to its constituents. Consumption of a 2% PEF‐supplemented HF diet significantly changed the mesenteric fat weight (*p* < 0.05) (Table 5). However, PEF‐supplemented HFD did not alter the HFD‐mediated increases in body weight and liver weight, as well as epididymal and peritoneal fat deposition. Similarly, consumption of a 2% PEF‐supplemented normal diet did not change any of these parameters, including mesenteric fat weight, significantly in comparison with the control diet group (Table 6).

**Table 6:** Effects of *Phyllanthus emblica* fruit powder supplementation on food consumption, body weight change, liver weight change, and different types of adipose tissue weights on HFD‐induced hyperlipidemic rats.

Parameters	Control	Control + PEF	HFD	HFD + PEF
Initial body weight (g)	194.56 ± 2.25^a^	193.25 ± 3.42^a^	191.56 ± 2.25^a^	192.33 ± 3.38^a^
Final body weight (g)	265.31 ± 5.36^a^	273.23 ± 6.92^a^	316.23 ± 6.24^b^	308.23 ± 5.96^b^
Food intake (g/rat/day)	19.23 ± 2.51^a^	21.5 ± 3.12^a^	19.17 ± 2.64^a^	20.27 ± 2.45^a^
Liver weight (g/100 g body weight)	3.51 ± 0.43^a^	3.35 ± 0.41^a^	4.83 ± 0.63^b^	4.43 ± 0.54^b^
Epididymal fat (g/100 g body weight)	1.13 ± 0.17^a^	1.22 ± 0.18^a^	1.85 ± 0.21^b^	1.68 ± 0.17^b^
Mesenteric fat (g/100 g body weight)	1.07 ± 0.11^a^	1.05 ± 0.11^a^	1.47 ± 0.13^b^	1.12 ± 0.12^a^
Peritoneal fat (g/100 g body weight)	0.78 ± 0.09^a^	0.83 ± 0.12^a^	1.29 ± 0.16^b^	1.22 ± 0.08^b^

*Note:* The data shown here are the average of 6 values obtained from the samples (*n* = 6) of a particular group. For statistical comparison, values were analyzed in “one‐way ANOVA” which was followed by the Newman–Keuls post hoc test. Significantly (*p* < 0.05) different values within the same row are represented by different superscript letters. Four groups of rats were provided with any one of the four diets: normal diet (Control), control diet containing 2% (w/w) dried powder of *P. emblica* fruit (Control + PEF), high‐fat diet (HFD), or HFD containing 2% PEF (HFD + PEF).

### 3.6. Effect of PEF on HFD‐Induced Oxidative Stress

**Figure 5:**
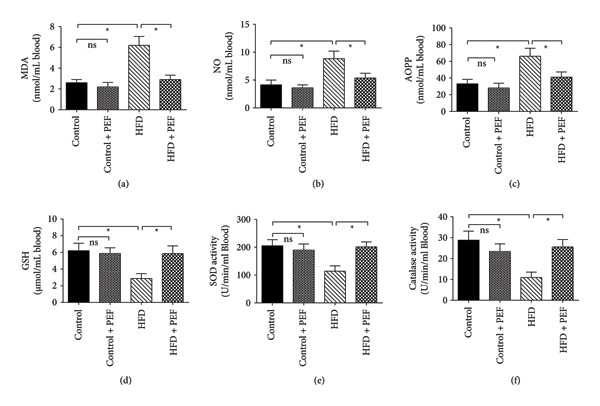
Effect of 2% (w/w) dried powder of *P. emblica* fruit (PEF) supplementation either with normal diet or with high‐fat diet on oxidative stress parameters such as MDA (a), NO (b), AOPP (c), GSH (d), SOD activity (e), and catalase activity (f). The rats were provided with any one of the four diets: normal diet (Control), control diet containing 2% (w/w) dried powder of *P. emblica* fruit (Control + PEF), high‐fat diet (HFD), or HFD containing 2% PEF (HFD + PEF). Values are shown as mean ± SEM, *n* = 6. For comparison, one‐way ANOVA was conducted which was followed by Newman–Keuls multiple comparison tests. Mean values are considered significantly different when *p* < 0.05, which is represented by an asterisk (^∗^).

Due to HFD feeding, the plasma concentrations of MDA, nitric oxide (NO), and AOPP increased markedly (*p* < 0.05). On the other hand, the level of endogenous antioxidants, GSH, and the activity of antioxidant enzymes such as SOD and catalase were significantly decreased by HFD feeding. Feeding 2% PEF‐supplemented HFD resulted in a marked (*p* < 0.05) augmentation of the activity of SOD and catalase. Consequently, the reduction in oxidative stress was revealed as a significant lowering of the levels of MDA, NO, and AOPP. Additionally, HFD‐mediated depletion of GSH was replenished markedly (*p* < 0.05) due to PEF feeding. Similar to the previous cases, consumption of 2% PEF with a normal diet did not affect these oxidative stress parameters, indicating the efficacy of PEF supplementation only under stressful conditions (Figure 5).

### 3.7. Hypolipidemic and Hypoglycemic Effects of *P. emblica* Fruit

To explore the hypolipidemic and hypoglycemic effects of the PEF‐supplemented diet, triglyceride (TG), TC, LDLC, HDLC, and FBS levels were measured. Consumption of HFD led to a marked increase in all of these parameters, except HDLC, which was reduced in HFD‐consuming rats. Feeding of 2% (w/w) PEF‐supplemented HFD significantly (*p* < 0.05) reduced the levels of TC, LDLC, TG, and FBS. In contrast, the HFD‐suppressed level of HDLC in the plasma was restored by the addition of 2% PEF powder in the HFD. However, consumption of 2% PEF‐supplemented standard diet (Control + PEF) could not change these parameters significantly (Table 7).

**Table 7:** Effect of *Phyllanthus emblica* fruit powder supplementation on the lipid profile and fasting blood sugar (FBS) of high‐fat diet‐consuming rats.

Parameter	Control	Control + PEF	HFD	HFD + PEF
TG (mg/dL)	139.2 ± 12.5^a^	129.3 ± 13.2^a^	196.3 ± 17.8^b^	143.4 ± 15.6^a^
TC (mg/dL)	192.4 ± 23.1^a^	195.7 ± 21.6^a^	265.4 ± 27.5^b^	202.5 ± 19.7^a^
HDLC (mg/dL)	67.5 ± 9.2^a^	62.8 ± 7.5^a^	43.3 ± 5.1^b^	69.5 ± 9.8^a^
LDLC (mg/dL)	96.8 ± 8.1^a^	93.7 ± 11.2^a^	173.8 ± 15.6^b^	117.5 ± 15.1^a^
FBS (mg/dL)	72.3 ± 5.7^a^	76.3 ± 5.1^a^	141.8 ± 10.3^b^	82.8 ± 9.3^a^

*Note:* Four groups of rats were provided with any one of the four diets: normal diet (control), control diet containing 2% (w/w) dried powder of *P. emblica* fruit (control + PEF), high‐fat diet (HFD), or HFD containing 2% PEF (HFD + PEF). The data shown here are the average of 6 values obtained from the samples (*n* = 6) of a particular group. For statistical comparison, values were analyzed in “one‐way ANOVA” which was followed by the Newman–Keuls post hoc test. Significantly (*p* < 0.05) different values within the same row are represented by different superscript letters.

### 3.8. Effects of *P. emblica* on the Expression of Target Genes

**Figure 6:**
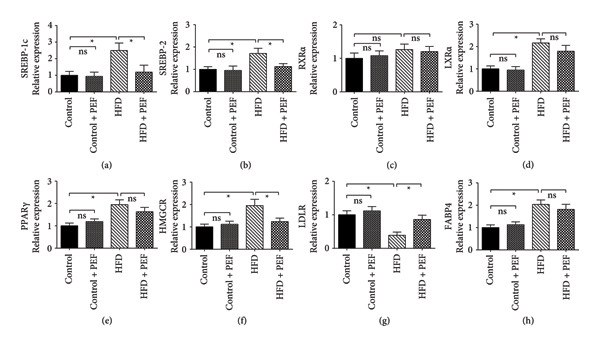
Effect of 2% (w/w) dried powder of *P. emblica* fruit (PEF) supplementation either with normal diet or with high‐fat diet on adipogenesis controlling transcription factors, enzymes, and receptor proteins. The rats were provided with any one of the four diets: normal diet (control), control diet containing 2% (w/w) dried powder of *P. emblica* fruit (Control + PEF), high‐fat diet (HFD), or HFD containing 2% PEF (HFD + PEF). After 8 weeks of feeding, the rats were sacrificed and mRNA was extracted from the hepatic tissue, followed by mRNA measurement and cDNA synthesis using 1 μg mRNA from each sample. Specific primers and a quantitative PCR system were used to quantify the mRNA levels of the corresponding genes, which were normalized to the mRNA levels of β‐actin. Values are shown as mean ± SEM, *n* = 6. For comparison, one‐way ANOVA was conducted which was followed by Newman–Keuls multiple comparison tests. Mean values are considered significantly different when *p* < 0.05, which is represented by an asterisk (^∗^).

Feeding with an HFD substantially (*p* < 0.05) augmented the gene expression of SREBP‐1c and SREBP‐2, LXRα, PPARγ, HMGCR, and FABP4. However, the gene expression of RXRα was not changed and the expression of LDL receptor (LDLR) was downregulated by HFD feeding. The consumption of 2% PEF with HFD downregulated the transcript levels of SREBP‐1c, SREBP‐2, and HMGCR compared with the HFD‐fed rats. On the other hand, the HFD‐mediated suppression of LDLR was restored by treatment with PEF together with HFD. However, the HFD‐mediated enhancement of the mRNA levels of PPARγ, LXRα, and FABP4 remained unchanged by the feeding of PEF (Figure 6). The expression of the aforementioned proteins remained similar both in the control and in 2% PEF‐supplemented control diet groups (Figure 6).

### 3.9. Effects of *Phyllanthus emblica* on Hepatic Fat Accumulation

The hypolipidemic effect of the PEF‐supplemented diet was also evaluated by H&E staining of hepatic tissues. As revealed in the photomicrographs, the number and size of lipid droplets (LDs) increased markedly (*p* < 0.05) because of the consumption of an HFD. Feeding a PEF‐supplemented diet did not change the number and size of LDs (Figure 7), suggesting the validity of a nonsignificant reduction in liver weight due to the consumption of PEF‐supplemented HFD (Table 6). The photomicrographs were also analyzed using ImageJ software, which converts the changes into quantitative data suitable for statistical comparisons. Similar to the photomicrographs, feeding with PEF‐supplemented HFD did not alter the HFD‐induced lipid accumulation in the liver (Figure 7(e)).

Figure 7Photomicrographs (a–d) displaying the accumulation of lipid in the hepatic tissue of Wistar rats that were fed with any one of the following diets: normal diet (Control), control diet containing 2% (w/w) dried powder of *P. emblica* fruit (Control + PEF), high‐fat diet (HFD), or HFD containing 2% PEF (HFD + PEF). Lipid droplets (LDs) were captured in 40× magnification by an optical microscope (Carl Zeiss). Additionally, the percentage of the area taken up by the lipid droplets (e) was measured using ImageJ software. To determine the statistical significance, the percent area was compared in one‐way ANOVA followed by Newman–Keuls’s post hoc test and expressed as mean ± SEM (*n* = 3).

**Figure:**
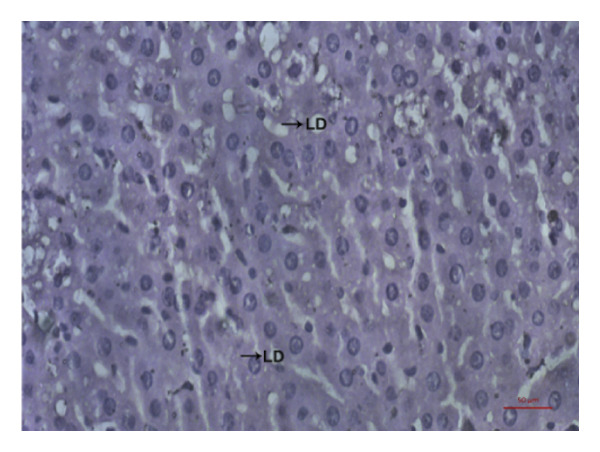
(a)

**Figure:**
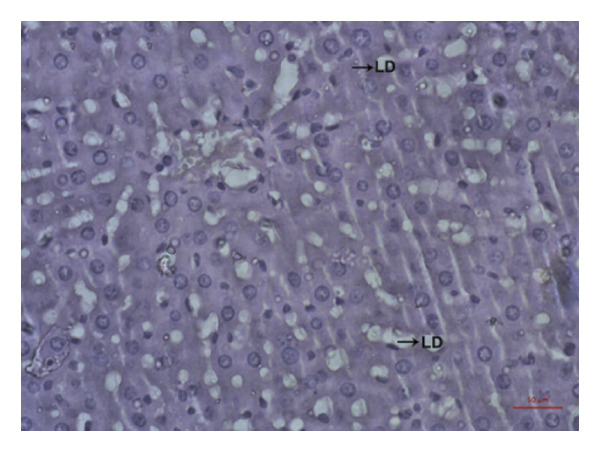
(b)

**Figure:**
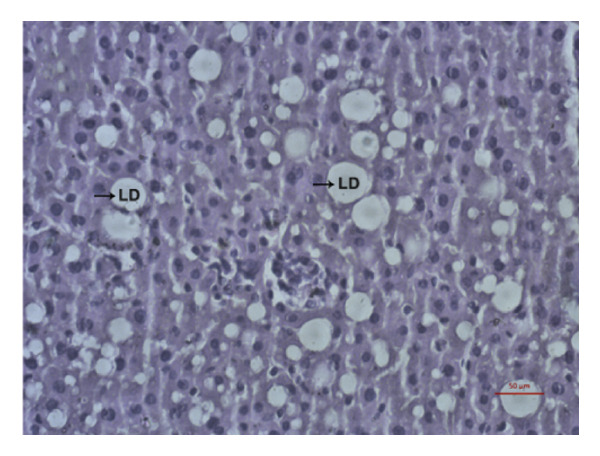
(c)

**Figure:**
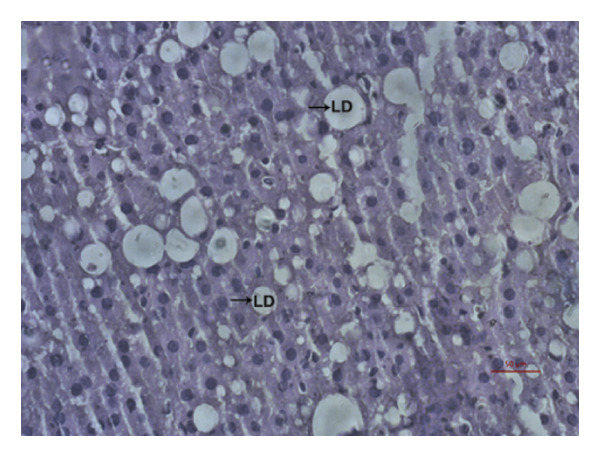
(d)

**Figure:**
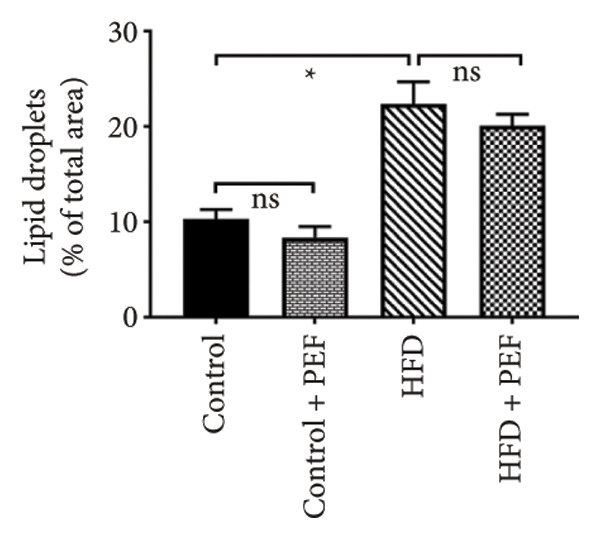
(e)

## 4. Discussion

Hyperlipidemia involves the activation of signal transduction mechanisms triggered by several transcription factors. Among these factors, SREBP‐1c and SREBP‐2, RXRα, LXRα, and PPARγ are of pivotal importance [29, 57]. These factors control the expression of some enzymes and receptors such as HMGCR, LDLR, and FABP4, which have crucial roles in lipid metabolism, homeostasis, and storage [58–60]. These enzymes and receptors and their regulatory factors are strongly modulated by oxidative stress and HFD consumption [61]. An HFD induces oxidative stress and lipid metabolism abnormalities due to HFD‐mediated suppression of SOD, catalase, and GPx, leading to reduced peroxide and superoxide degradation and consequent increase in ROS levels [62, 63]. This increased level of ROS intensifies chronic inflammation and lipid metabolism abnormalities, leading to hyperlipidemia. The interplay between hyperlipidemia and oxidative stress creates a self‐perpetuating pathogenic loop that leads to several cardiovascular diseases [64]. Scientific innovations have enabled the discovery and synthesis of antihyperlipidemic drugs. However, their therapeutic benefits are often compromised by harmful side effects. As a result, there has been a growing body of research focusing on screening medicinal plants, aimed at identifying phytochemicals with antioxidant and lipid‐lowering properties.

Numerous natural compounds, especially antioxidant‐type phytochemicals, have been investigated in the last few decades to identify potential lipid‐lowering molecules [65]. The fruit pulp of *P. emblica* contains a substantial amount of vitamins, tannins, saponins, phenolics, flavonoids, and alkaloids; many of which possess remarkable antioxidant activity [66]. In the present investigation, our results demonstrated that the antioxidant capacity of *P. emblica* fruit was significantly (*p* < 0.05) higher than that of BHT, although it was lower than that of ascorbic acid (Table 4). This improved antioxidant activity is likely attributable to the presence of several phenolic compounds and flavonoids of PEF, as was confirmed by HPLC analysis (Figure 4). In agreement with our observation, researchers through HPLC, GC‐MS, and LC‐MS techniques confirmed the presence of gallic acid, ellagic acid, quercetin, chrysin, myricitrin, myricetin, kaempferol, ferulic acid, cinnamic acid, caffeic acid, coumaric acid, quinic acid, and catechin in the fruit of *Emblica officinalis* and *Phyllanthus emblica* [9, 10, 67]. Studies have also confirmed the *in vitro* and *in vivo* antioxidant properties and the health benefits of these phenolic compounds [68]. Therefore, we predicted that a phenolic and flavonoid‐rich PEF‐supplemented diet may also suppress oxidative stress *in vivo*, as it does *in vitro* (Table 4). Subsequently, in our animal study, we also observed that a PEF‐enriched diet restored the depleted level of GSH with a simultaneous increase in the activities of catalase and SOD. The enhanced antioxidant capacity of PEF supplementation was reflected by the reduced concentrations of MDA, nitric oxide (NO), and AOPP (Figure 5). The aforementioned studies and our observations suggest the importance of exploring the molecular mechanisms of these phytochemicals to understand their roles in HFD‐induced oxidative stress and hyperlipidemia. However, very little has been done in this regard, and therefore, the molecular mechanism of these beneficial phytochemicals remains poorly understood.

In understanding the molecular mechanisms of various phytochemicals, the use of computer‐aided drug design has impressively supported the ongoing efforts of discovering novel, safe, and effective drug‐like lead compounds for many ailments. The combination of *in silico* approaches with *in vitro* and *in vivo* empirical knowledge about natural products has made drug discovery and development processes faster and less expensive [69]. In this study, through a literature survey, 21 compounds of *P. emblica* were selected for *in silico* investigations based on their antioxidant, anti‐inflammatory, lipid‐lowering, and glucose‐lowering effects (Supporting Table 1). Out of those 21 compounds, 16 were found to possess acceptable bioavailability characteristics and drug‐likeness (Table 2). Moreover, two compounds, coumaric acid and chrysin, were able to cross the BBB (Figure 3), which may offer additional benefits by protecting the BBB or ameliorating HFD‐induced oxidative stress and neuroinflammatory damage. In agreement with our prediction, researchers have reported that coumaric acid increases BBB integrity by increasing the expression of occludin in hypoxic mice [70]. Similarly, chrysin ameliorates brain injury‐induced BBB disruption, reduces brain edema, and prevents neuron loss [71]. Additionally, other polyphenols of PEF, including quercetin, kaempferol, and rutin, contribute to the protection of the BBB through their antioxidant and anti‐inflammatory properties [72]. Therefore, molecular docking studies were conducted on these 16 compounds. By considering binding affinities determined by molecular docking studies, we observed that 5 phytochemicals of *P. emblica*, such as quercetin, kaempferol, catechin, ellagic acid, and chrysin, potentially interact with 8 important proteins strongly associated with hyperlipidemia (Table 3). These proteins include SREBP‐1c, SREBP‐2, RXRα, LXRα, PPARγ, HMGCR, LDLR, and FABP4. Moreover, these proteins also interacted with the remaining 11 phytochemicals of PEF (Table 3). These observations obtained from the *in silico* study suggest that the already reported antihyperlipidemic and antihyperglycemic activity of *P. emblica* fruit can be attributed to the possible interaction of its phytochemicals with the aforementioned eight proteins. However, *in silico* predictions should be validated by *in vivo* investigations at the transcriptional level to decipher the mechanism of those phytochemicals’ efficacy in regulating lipid metabolism, transport, and storage.

Therefore, the transcript levels of the aforementioned eight proteins were measured using qPCR to elucidate the mechanism through which the phytochemicals of PEF affect lipid metabolism. We observed that transcript levels of SREBP‐1c, SREBP‐2, LXRα, PPARγ, HMGCR, and FABP4 increased in HFD‐fed rats. These increased expressions were logically connected to the increased levels of plasma lipids, such as TC, LDLC, and TG. Moreover, increased fat accumulation in the adipose and hepatic tissues contributed to the overall gain of body and liver weight in HFD‐consuming rats. Our findings align with those of previous studies in which feeding with HFD increased the harmful blood lipids and body weight in experimental animals by upregulating the expression of SREBP‐1c, SREBP‐2, LXRα, PPARγ, and FABP4 [73–75]. PEF‐supplemented HFD significantly (*p* < 0.05) lowered the expression of SREBP‐1c, SREBP‐2, and HMGCR compared with HFD‐fed rats (Figure 6). Our findings were in agreement with a study in which Murase et al. observed that catechin‐containing coffee suppressed diet‐induced hypertriglyceridemia in mice through the suppression of SREBP‐1c [76]. Moreover, our observations are in agreement with previous studies in which researchers observed that quercetin reduces hypercholesterolemia and lipid accumulation in adipose tissues through the suppression of SREBP‐2 and HMGCR [59, 77].

In the regulation of lipid metabolism and homeostasis, SREBPs also closely work with LXRα and PPARγ [78]. Considering this interconnection among these crucial transcription factors, we predicted that the HFD‐mediated increased expression of LXRα and PPARγ might be altered by PEF supplementation. However, the gene expression of PPARγ and LXRα was not changed significantly by PEF supplementation compared with the HFD‐fed rats (Figure 6). This inefficacy of PEF in suppressing the expression of PPARγ and LXRα is consistently reflected in the insignificant reduction of epididymal and peritoneal fat weight (Table 6), as well as liver fat accumulation (Figure 7), although the amount of mesenteric fat was reduced significantly (Table 6). Thus, our observation was in agreement with the result of a previous investigation, which demonstrated that increased expression of PPARγ and LXRα is crucial for fat deposition in cultured 3T3‐L1 cells and human adipocytes [79]. This could be, at least partially, due to quercetin in PEF. Feeding of mice with quercetin upregulated LXRα and PPARγ expression in hepatocytes and consequently increased fat accumulation in the liver [80, 81]. Additionally, according to other studies, flavonoids such as quercetin and kaempferol by activating LXRα and PPARγ upregulate the expression of cholesterol efflux proteins such as ATP‐binding cassette transporters (ABCA1 and ABCG1) and Apo‐A1, the constituent protein of HDL particles [82, 83]. Thus, flavonoids, by increasing the expression of cholesterol efflux proteins and HDL particle’s shell protein, elevate the levels of HDLC in the circulation [84].

In contrast to other proteins, HFD‐mediated suppression of LDLR was upregulated significantly due to the feeding of PEF. According to previous reports, flavonoids such as kaempferol and quercetin can upregulate the expression of LDLRs, leading to increased cellular uptake of LDLC from the circulation [85, 86]. This part of our study also complies with a previous investigation in which researchers reported that ellagic acid, quercetin, and myricetin‐containing extract of *Basella alba*–inhibited HMGCR but stimulated LDLR activity [87]. Hence, the increased expression of LDLR due to PEF feeding might be attributable to its phenolic compounds (Figure 4). Thus, a phenolic and flavonoid‐rich PEF‐supplemented diet by maintaining the upregulated state of PPARγ and LXRα, helped to retain harmful lipids in fat storage, limiting their presence in the circulation [88]. On the other hand, the PEF‐supplemented diet by suppressing the expression of SREBP‐1c, SREBP‐2, and HMGCR, along with the upregulation of LDLR, reduced the levels of harmful lipids in the blood. Therefore, we concluded that although 2% PEF supplementation for 8 weeks effectively reduced HFD‐induced hyperlipidemia, it did not reduce fat storage and consequently body weight. However, the administration of higher doses (> 2%) of PEF for a longer time (> 8 weeks) may result in the reduction of both adipose tissue mass and hyperlipidemia, indicating the need for further investigation.

## 5. Conclusion

The dried powder of PEF, used in the current study, contains a number of bioactive phenolic compounds, such as gallic acid, ellagic acid, quercetin, kaempferol, catechin, and chrysin among which the latter four compounds are also flavonoid. These compounds possess antioxidant properties, which are further supported by their *in vitro* antioxidant and radical scavenging activities as well as *in vivo* suppression of oxidative stress. *In silico* prediction analysis revealed that these compounds of PEF are bioavailable after oral administration and can interact with several transcription factors, enzymes, and receptors that are strongly connected to lipid metabolism and homeostasis. Through its higher phenolic content and strong antioxidant properties, PEF downregulated the expression of SREBP‐1c, SREBP‐2, and HMGCR, along with the upregulation of LDLR expression. All these effects culminated in the reduced concentration of harmful lipids in the blood. Despite the lowering of harmful plasma lipid levels, body weight, adipose tissue weight, and liver fat accumulation were not reduced significantly. This outcome justifies the inefficacy of PEF in modulating the expression of LXRα and PPARγ. Polyphenolics‐mediated augmented expression of these factors helped not only to maintain higher levels of HDLC but also to retain harmful lipids in storage locations, preventing hyperlipidemia. Taking all these observations together, we concluded that PEF‐mediated suppression of gene expression of SREBP‐1c and SREBP‐2 and consequent suppression of HMGCR along with upregulation of LDLR played central roles in ameliorating HFD‐induced hyperlipidemia in Wistar rats. The bioactive constituents of *P. emblica* fruit such as ellagic acid, quercetin, kaempferol, chrysin, and catechin can be the starting lead compounds with a high possibility for the development of antihyperlipidemic drugs with specific targeted pharmacological activities and minimal adverse effects [89–92].

## Disclosure

All authors have read and approved the final manuscript.

## Conflicts of Interest

The authors declare no conflicts of interest.

## Author Contributions

Syed Abdul Kuddus: experimental design, investigation, in silico study, and data analysis. Md. Hasanuzzaman Shohag: investigation and HPLC analysis. Quamrul Islam Yaseen: animal feeding and investigation. Labiba Ahmed: animal feeding and investigation. Anika Tabassum Kashfia: animal feeding and investigation. Fatema Binte Islam: animal feeding and investigation. Sabrin Islam Khan: investigation and phytochemical assay. Reatul Karim: experimental design, writing, and editing. Muhammad Maqsud Hossain: RNA isolation and quantitative real‐time PCR. Md. Ashraful Alam: conceptualization, experimental design, and data analysis. Ferdous Khan: conceptualization, experimental design, data analysis, writing–original draft, and supervision.

## Funding

This project was funded by the North South University Research Grant Cycle 2019‐20, allocated for Ferdous Khan (ID: CTRG‐19/SHLS/24).

## Supporting Information

Supporting Figure: 1. Gallic acid, 2. quinic acid, 3. leucine, 4. malic acid, 5. catechin, 6. coumaric acid, 7. betaine, 8. trigonelline, 9. ellagic acid, 10. caffeic acid, 11. chrysin, 12. myricetin, 13. methyl gallate, 14. kaempferol, 15. quercetin, and 16. betulinic acid.

Supporting Table 1: Pharmacological activities of several compounds present in the *Phyllanthus emblica* fruit pulp.

Supporting Table 2: Composition of the normal diet (Control), the HFD, normal diet supplemented with 2% (w/w) dried powder of *P. emblica* fruit (Control + PEF), and HFD supplemented with 2% (w/w) dried powder of *P. emblica* fruit (HFD + PEF).

Supporting Table 3: Cytochrome enzyme inhibitory activities, lead‐likeness, and synthetic accessibility of selected compounds of *P. emblica* fruit predicted by SWISS prediction tool.

## Supporting information


**Supporting Information** Additional supporting information can be found online in the Supporting Information section.

## Data Availability

All the data and information generated and scrutinized in this investigation are incorporated in this manuscript and its supporting information file.

## References

[1] Halpern A. , Mancini M. C. , Magalhães M. E. C. et al., Metabolic Syndrome, Dyslipidemia, Hypertension and Type 2 Diabetes in Youth: From Diagnosis to Treatment, Diabetology & Metabolic Syndrome. (2010) 2, no. 1, 1–20, 10.1186/1758-5996-2-55, 2-s2.0-77956692453. 20718958 PMC2939537

[2] Hill M. F. and Bordoni B. , Hyperlipidemia, StatPearls [Internet]. (2022) StatPearls Publishing, Treasure Island, FL.

[3] Hedayatnia M. , Asadi Z. , Zare-Feyzabadi R. et al., Dyslipidemia and Cardiovascular Disease Risk Among the MASHAD Study Population, Lipids in Health and Disease. (2020) 19, 1–11, 10.1186/s12944-020-01204-y. 32178672 PMC7075010

[4] Nelson R. H. , Hyperlipidemia as a Risk Factor for Cardiovascular Disease, Primary Care: Clinics in Office Practice. (2013) 40, no. 1, 195–211, 10.1016/j.pop.2012.11.003, 2-s2.0-84873586706. 23402469 PMC3572442

[5] Zodda D. , Giammona R. , and Schifilliti S. , Treatment Strategy for Dyslipidemia in Cardiovascular Disease Prevention: Focus on Old and New Drugs, Pharmacy. (2018) 6, no. 1, 10.3390/pharmacy6010010. PMC5874549 29361723

[6] Ahmed K. , Jahan I. , Jahan F. , and Hossain H. , Antioxidant Activities and Simultaneous HPLC-DAD Profiling of Polyphenolic Compounds From *Moringa oleifera* Lam. Leaves Grown in Bangladesh, Food Research. (2021) 5, no. 1, 401–408, 10.26656/fr.2017.5(1).410.

[7] Gul M. , Liu Z.-W. , Rabail R. et al., Functional and Nutraceutical Significance of Amla (*Phyllanthus emblica* L.): A Review, Antioxidants. (2022) 11, no. 5, 10.3390/antiox11050816. PMC9137578 35624683

[8] Mirunalini S. and Krishnaveni M. , Therapeutic Potential of *Phyllanthus emblica* (Amla): The Ayurvedic Wonder, Journal of Basic and Clinical Physiology and Pharmacology. (2010) 21, no. 1, 93–105, 10.1515/jbcpp.2010.21.1.93, 2-s2.0-77953153977. 20506691

[9] Wu L. , Zhang Q. , Liang W. , Ma Y. , Niu L. , and Zhang L. , Phytochemical Analysis Using UPLC-MSn Combined With Network Pharmacology Approaches to Explore the Biomarkers for the Quality Control of the Anticancer Tannin Fraction of *Phyllanthus emblica* L. Habitat in Nepal, Evidence-Based Complementary and Alternative Medicine. (2021) 2021, 6623791–19, 10.1155/2021/6623791. 33833816 PMC8018855

[10] Gaire B. P. and Subedi L. , Phytochemistry, Pharmacology and Medicinal Properties of *Phyllanthus emblica* Linn, Chinese Journal of Integrative Medicine. (2014) 20, 1–8, 10.1007/s11655-014-1984-2. 25491539

[11] Li W. , Zhang X. , Chen R. et al., HPLC Fingerprint Analysis of *Phyllanthus emblica* Ethanol Extract and Their Antioxidant and Anti-Inflammatory Properties, Journal of Ethnopharmacology. (2020) 254, 10.1016/j.jep.2020.112740. 32151757

[12] Wu M. , Cai J. , Fang Z. et al., The Composition and Anti-Aging Activities of Polyphenol Extract From *Phyllanthus emblica* L. Fruit, Nutrients. (2022) 14, no. 4, 10.3390/nu14040857. PMC8878974 35215512

[13] Rahman M. M. , Ferdous K. U. , Roy S. et al., Polyphenolic Compounds of Amla Prevent Oxidative Stress and Fibrosis in the Kidney and Heart of 2K1C Rats, Food Science and Nutrition. (2020) 8, no. 7, 3578–3589, 10.1002/fsn3.1640. 32724620 PMC7382108

[14] Zhou Y. , Qian C. , Tang Y. et al., Advance in the Pharmacological Effects of Quercetin in Modulating Oxidative Stress and Inflammation Related Disorders, Phytotherapy Research. (2023) 37, no. 11, 4999–5016, 10.1002/ptr.7966. 37491826

[15] Ali M. , Hassan M. , Ansari S. A. , Alkahtani H. M. , Al-Rasheed L. S. , and Ansari S. A. , Quercetin and Kaempferol as Multi-Targeting Antidiabetic Agents Against Mouse Model of Chemically Induced Type 2 Diabetes, Pharmaceuticals. (2024) 17, no. 6, 10.3390/ph17060757. PMC11206732 38931424

[16] Xu Q. , Li S. , Tang W. et al., The Effect of Ellagic Acid on Hepatic Lipid Metabolism and Antioxidant Activity in Mice, Frontiers in Physiology. (2021) 12, 10.3389/fphys.2021.751501. PMC8529006 34690819

[17] Guo M. , Xie L. , Yuan H. , Liao D. F. , and Zheng X. L. , Catechin Inhibits ox-LDL-Induced Ferroptosis in Vascular Smooth Muscle Cells to Alleviate and Stabilize Atherosclerosis, Frontiers in Nutrition. (2025) 12, 10.3389/fnut.2025.1594708. PMC12171153 40529424

[18] Banerjee A. , Singh S. , Prasad S. K. et al., Protective Efficacy of *Tinospora sinensis* Against Streptozotocin Induced Pancreatic Islet Cell Injuries of Diabetic Rats and Its Correlation to Its Phytochemical Profiles, Journal of Ethnopharmacology. (2020) 248, 10.1016/j.jep.2019.112356. 31669668

[19] Biswas T. K. , Chakrabarti S. , Pandit S. , Jana U. , and Dey S. K. , Pilot Study Evaluating the Use of *Emblica officinalis* Standardized Fruit Extract in Cardio-Respiratory Improvement and Antioxidant Status of Volunteers With Smoking History, Journal of Herbal Medicine. (2014) 4, no. 4, 188–194, 10.1016/j.hermed.2014.09.002, 2-s2.0-84940164651.

[20] Brown P. D. , Ketter N. , Vis-Dunbar M. , and Sakakibara B. M. , Clinical Effects of *Emblica officinalis* Fruit Consumption on Cardiovascular Disease Risk Factors: A Systematic Review and Meta-Analysis, BMC Complementary Medicine and Therapies. (2023) 23, no. 1, 10.1186/s12906-023-03997-8. PMC10251691 37296402

[21] Usharani P. , Merugu P. L. , and Nutalapati C. , Evaluation of the Effects of a Standardized Aqueous Extract of *Phyllanthus emblica* Fruits on Endothelial Dysfunction, Oxidative Stress, Systemic Inflammation and Lipid Profile in Subjects With Metabolic Syndrome: A Randomised, Double Blind, Placebo Controlled Clinical Study, BMC Complementary and Alternative Medicine. (2019) 19, 1–8, 10.1186/s12906-019-2509-5, 2-s2.0-85065649389. 31060549 PMC6503348

[22] Huang C.-Z. , Tung Y.-T. , Hsia S.-M. , Wu C.-H. , and Yen G.-C. , The Hepatoprotective Effect of *Phyllanthus emblica* L. Fruit on High Fat Diet-Induced Non-Alcoholic Fatty Liver Disease (NAFLD) in SD Rats, Food & Function. (2017) 8, no. 2, 842–850, 10.1039/c6fo01585a, 2-s2.0-85013918433. 28128372

[23] Maiti S. , Chattopadhyay S. , Acharyya N. , Deb B. , and Hati A. K. , *Emblica officinalis* (Amla) Ameliorates Arsenic-Induced Liver Damage via DNA Protection by Antioxidant Systems, Molecular & Cellular Toxicology. (2014) 10, no. 1, 75–82, 10.1007/s13273-014-0009-8, 2-s2.0-84901228622.

[24] Nain P. , Saini V. , Sharma S. , and Nain J. , Antidiabetic and Antioxidant Potential of *Emblica officinalis* Gaertn. Leaves Extract in Streptozotocin-Induced Type-2 Diabetes Mellitus (T2DM) Rats, Journal of Ethnopharmacology. (2012) 142, no. 1, 65–71, 10.1016/j.jep.2012.04.014, 2-s2.0-84861981815. 22855943

[25] Singh M. K. , Yadav S. S. , Gupta V. , and Khattri S. , Immunomodulatory Role of *Emblica officinalis* in Arsenic Induced Oxidative Damage and Apoptosis in Thymocytes of Mice, BMC Complementary and Alternative Medicine. (2013) 13, 1–13, 10.1186/1472-6882-13-193, 2-s2.0-84880954801. 23889914 PMC3733846

[26] Chahal A. K. , Chandan G. , Kumar R. , Chhillar A. K. , Saini A. K. , and Saini R. V. , Bioactive Constituents of *Emblica officinalis* Overcome Oxidative Stress in Mammalian Cells by Inhibiting Hyperoxidation of Peroxiredoxins, Journal of Food Biochemistry. (2020) 44, no. 2, 10.1111/jfbc.13115. 31821595

[27] Yamamoto H. , Morino K. , Mengistu L. et al., Amla Enhances Mitochondrial Spare Respiratory Capacity by Increasing Mitochondrial Biogenesis and Antioxidant Systems in a Murine Skeletal Muscle Cell Line, Oxidative Medicine and Cellular Longevity. (2016) 2016, 1735841–11, 10.1155/2016/1735841, 2-s2.0-84975260030. 27340504 PMC4909908

[28] Zhang Y. , Zhao L. , Guo X. et al., Chemical Constituents From *Phyllanthus emblica* and the Cytoprotective Effects on H_2_O_2_-Induced PC12 Cell Injuries, Archives of Pharmacal Research. (2016) 39, no. 9, 1202–1211, 10.1007/s12272-014-0433-2, 2-s2.0-84940285701. 24993870

[29] Alves M. , Laranjeira F. , and Correia-da-Silva G. , Understanding Hypertriglyceridemia: Integrating Genetic Insights, Genes. (2024) 15, no. 2, 10.3390/genes15020190. PMC10887881 38397180

[30] Lee J.-E. , Schmidt H. , Lai B. , and Ge K. , Transcriptional and Epigenomic Regulation of Adipogenesis, Molecular and Cellular Biology. (2019) 39, no. 11, e00601–e00618, 10.1128/mcb.00601-18, 2-s2.0-85066163950. 30936246 PMC6517598

[31] Macalino S. J. Y. , Gosu V. , Hong S. , and Choi S. , Role of Computer-Aided Drug Design in Modern Drug Discovery, Archives of Pharmacal Research. (2015) 38, no. 9, 1686–1701, 10.1007/s12272-015-0640-5, 2-s2.0-84941997753. 26208641

[32] Yu W. and MacKerell A. D. , Computer-Aided Drug Design Methods, Antibiotics: Methods and Protocols. (2017) Springer, Berlin, 85–106. 10.1007/978-1-4939-6634-9_5 PMC5248982 27873247

[33] Quranayati Q. , Iqhrammullah M. , Saidi N. , Nurliana N. , Idroes R. , and Nasution R. , Cytotoxicity and Phytochemical Profiles of *Phyllanthus emblica* Stem Barks With *In Silico* Drug-Likeliness: Focusing on Antidiabetic Potentials, Journal of Advanced Pharmaceutical Technology & Research. (2022) 13, no. 4, 281–285, 10.4103/japtr.japtr_319_22. 36568059 PMC9784048

[34] Sharma P. , Joshi T. , Joshi T. , Chandra S. , and Tamta S. , *In Silico* Screening of Potential Antidiabetic Phytochemicals From *Phyllanthus emblica* Against Therapeutic Targets of Type 2 Diabetes, Journal of Ethnopharmacology. (2020) 248, 10.1016/j.jep.2019.112268. 31593813

[35] Ursu O. , Rayan A. , Goldblum A. , and Oprea T. I. , Understanding Drug-Likeness, Wiley Interdisciplinary Reviews: Computational Molecular Science. (2011) 1, no. 5, 760–781, 10.1002/wcms.56, 2-s2.0-84962352796.

[36] Daina A. and Zoete V. , A Boiled‐Egg to Predict Gastrointestinal Absorption and Brain Penetration of Small Molecules, ChemMedChem. (2016) 11, no. 11, 1117–1121, 10.1002/cmdc.201600182, 2-s2.0-84973652282. 27218427 PMC5089604

[37] Madhavi Sastry G. , Adzhigirey M. , Day T. , Annabhimoju R. , and Sherman W. , Protein and Ligand Preparation: Parameters, Protocols, and Influence on Virtual Screening Enrichments, Journal of Computer-Aided Molecular Design. (2013) 27, no. 3, 221–234, 10.1007/s10822-013-9644-8, 2-s2.0-84880529288. 23579614

[38] Terefe E. M. and Ghosh A. , Molecular Docking, Validation, Dynamics Simulations, and Pharmacokinetic Prediction of Phytochemicals Isolated From *Croton dichogamus* Against the HIV-1 Reverse Transcriptase, Bioinformatics and Biology Insights. (2022) 16, 10.1177/11779322221125605. PMC9516429 36185760

[39] Lien E. , Boyle F. , Wrenn J. , Perry R. , Thompson C. , and Borzelleca J. , Comparison of AIN-76A and AIN-93G Diets: A 13 Week Study in Rats, Food and Chemical Toxicology. (2001) 39, no. 4, 385–392, 10.1016/s0278-6915(00)00142-3, 2-s2.0-0035073694. 11295485

[40] Speakman J. R. , Use of High-Fat Diets to Study Rodent Obesity as a Model of Human Obesity, International Journal of Obesity. (2019) 43, no. 8, 1491–1492, 10.1038/s41366-019-0363-7, 2-s2.0-85064050789. 30967607

[41] Chandra S. , Khan S. , Avula B. et al., Assessment of Total Phenolic and Flavonoid Content, Antioxidant Properties, and Yield of Aeroponically and Conventionally Grown Leafy Vegetables and Fruit Crops: A Comparative Study, Evidence-Based Complementary and Alternative Medicine. (2014) 2014, 253875–9, 10.1155/2014/253875, 2-s2.0-84899426182. 24782905 PMC3980857

[42] Phuyal N. , Jha P. K. , Raturi P. P. , and Rajbhandary S. , Total Phenolic, Flavonoid Contents, and Antioxidant Activities of Fruit, Seed, and Bark Extracts of *Zanthoxylum armatum* DC, The Scientific World Journal. (2020) 2020, 8780704–7, 10.1155/2020/8780704. 32256249 PMC7102453

[43] Szerlauth A. , Muráth S. , Viski S. , and Szilagyi I. , Radical Scavenging Activity of Plant Extracts From Improved Processing, Heliyon. (2019) 5, no. 11, 10.1016/j.heliyon.2019.e02763. PMC6895678 31844703

[44] Vijayalakshmi M. and Ruckmani K. , Ferric Reducing Anti-Oxidant Power Assay in Plant Extract, Bangladesh Journal of Pharmacology. (2016) 11, no. 3, 570–572, 10.3329/bjp.v11i3.27663, 2-s2.0-84973457949.

[45] Hazra B. , Biswas S. , and Mandal N. , Antioxidant and Free Radical Scavenging Activity of *Spondias pinnata* , BMC Complementary and Alternative Medicine. (2008) 8, no. 1, 1–10, 10.1186/1472-6882-8-63, 2-s2.0-61349126555. 19068130 PMC2636748

[46] Ahloy-Dallaire J. , Klein J. D. , Davis J. K. , and Garner J. P. , Automated Monitoring of Mouse Feeding and Body Weight for Continuous Health Assessment, Laboratory Animals. (2019) 53, no. 4, 342–351, 10.1177/0023677218797974, 2-s2.0-85059520096. 30286683

[47] Rotondo F. , del Mar Romero M. , Ho-Palma A. C. , Remesar X. , Fernández-López J. A. , and Alemany M. , Quantitative Analysis of Rat Adipose Tissue Cell Recovery, and Non-Fat Cell Volume, in Primary Cell Cultures, PeerJ. (2016) 4, 10.7717/peerj.2725, 2-s2.0-85003815060. PMC5131620 27917316

[48] Zeb A. and Ullah F. , A Simple Spectrophotometric Method for the Determination of Thiobarbituric Acid Reactive Substances in Fried Fast Foods, Journal of Analytical Methods in Chemistry. (2016) 2016, 9412767–5, 10.1155/2016/9412767, 2-s2.0-84971384450. 27123360 PMC4830699

[49] Ghasemi A. , Hedayati M. , and Biabani H. , Protein Precipitation Methods Evaluated for Determination of Serum Nitric Oxide End Products by the Griess Assay, Journal of Medical Sciences Research. (2007) 2, no. 15, 29–32.

[50] Witko-Sarsat V. , Friedlander M. , Capeillère-Blandin C. et al., Advanced Oxidation Protein Products as a Novel Marker of Oxidative Stress in Uremia, Kidney International. (1996) 49, no. 5, 1304–1313, 10.1038/ki.1996.186, 2-s2.0-0029948839. 8731095

[51] Rahman I. , Kode A. , and Biswas S. K. , Assay for Quantitative Determination of Glutathione and Glutathione Disulfide Levels Using Enzymatic Recycling Method, Nature Protocols. (2006) 1, no. 6, 3159–3165, 10.1038/nprot.2006.378, 2-s2.0-34548851693. 17406579

[52] Kakkar P. , Das B. , and Viswanathan P. , A Modified Spectrophotometric Assay of Superoxide Dismutase, Indian Journal of Biochemistry & Biophysics. (1984) 21, no. 2, 130–132. 6490072

[53] Hadwan M. H. , Simple Spectrophotometric Assay for Measuring Catalase Activity in Biological Tissues, BMC Biochemistry. (2018) 19, no. 1, 1–8, 10.1186/s12858-018-0097-5, 2-s2.0-85051092311. 30075706 PMC6091033

[54] Khan F. , Syeda P. K. , Nartey M. N. N. et al., Pretreatment of Cultured Preadipocytes With Arachidonic Acid During the Differentiation Phase Without a cAMP-Elevating Agent Enhances Fat Storage After the Maturation Phase, Prostaglandins & Other Lipid Mediators. (2016) 123, 16–27, 10.1016/j.prostaglandins.2016.02.003, 2-s2.0-84962257446. 26928048

[55] Yaligar J. , Gopalan V. , Kiat O. W. et al., Evaluation of Dietary Effects on Hepatic Lipids in High Fat and Placebo Diet Fed Rats by *In Vivo* MRS and LC-MS Techniques, PLoS One. (2014) 9, no. 3, 10.1371/journal.pone.0091436, 2-s2.0-84898639621. PMC3956606 24638096

[56] Antypenko L. , Shabelnyk K. , Antypenko O. et al., *In Silico* Identification and Characterization of Spiro[1,2,4]Triazolo[1,5-c]Quinazolines as Diacylglycerol Kinase α Modulators, Molecules. (2025) 30, no. 11, 10.3390/molecules30112324. PMC12156019 40509212

[57] Liu R. , Liu X. , Bai X. , Xiao C. , and Dong Y. , Different Expression of Lipid Metabolism-Related Genes in Shandong Black Cattle and Luxi Cattle Based on Transcriptome Analysis, Scientific Reports. (2020) 10, no. 1, 1–14, 10.1038/s41598-020-79086-4. 33318614 PMC7736358

[58] Furuhashi M. , Saitoh S. , Shimamoto K. , and Miura T. , Fatty Acid-Binding Protein 4 (FABP4): Pathophysiological Insights and Potent Clinical Biomarker of Metabolic and Cardiovascular Diseases, Clinical Medicine Insights: Cardiology. (2014) 8, 10.4137/cmc.s17067, 2-s2.0-84981738067. PMC4315049 25674026

[59] Ness G. C. and Chambers C. M. , Feedback and Hormonal Regulation of Hepatic 3-Hydroxy-3-Methylglutaryl Coenzyme A Reductase: The Concept of Cholesterol Buffering Capacity (44508), Proceedings of the Society for Experimental Biology and Medicine. (2000) 224, no. 1, 8–19, 10.1046/j.1525-1373.2000.22359.x. 10782041

[60] Pal S. , Ho N. , Santos C. et al., Red Wine Polyphenolics Increase LDL Receptor Expression and Activity and Suppress the Secretion of ApoB100 From Human HepG2 Cells, The Journal of nutrition. (2003) 133, no. 3, 700–706, 10.1093/jn/133.3.700. 12612140

[61] Shoucri B. M. , Hung V. T. , Chamorro-García R. , Shioda T. , and Blumberg B. , Retinoid X Receptor Activation During Adipogenesis of Female Mesenchymal Stem Cells Programs a Dysfunctional Adipocyte, Endocrinology. (2018) 159, no. 8, 2863–2883, 10.1210/en.2018-00056, 2-s2.0-85050968145. 29860300 PMC6669823

[62] Lasker S. , Rahman M. M. , Parvez F. et al., High-Fat Diet-Induced Metabolic Syndrome and Oxidative Stress in Obese Rats Are Ameliorated by Yogurt Supplementation, Scientific Reports. (2019) 9, no. 1, 1–15, 10.1038/s41598-019-56538-0. 31882854 PMC6934669

[63] Yida Z. , Imam M. U. , Ismail M. , Ismail N. , Ideris A. , and Abdullah M. A. , High Fat Diet-Induced Inflammation and Oxidative Stress Are Attenuated by N-Acetylneuraminic Acid in Rats, Journal of Biomedical Science. (2015) 22, 1–10, 10.1186/s12929-015-0211-6, 2-s2.0-84944711697. 26498218 PMC4619312

[64] Alfadda A. A. and Sallam R. M. , Reactive Oxygen Species in Health and Disease, Journal of Biomedicine and Biotechnology. (2012) 2012, 936486–14, 10.1155/2012/936486, 2-s2.0-84866133072. 22927725 PMC3424049

[65] Goñi I. and Hernández-Galiot A. , Intake of Nutrient and Non-Nutrient Dietary Antioxidants. Contribution of Macromolecular Antioxidant Polyphenols in an Elderly Mediterranean Population, Nutrients. (2019) 11, no. 9, 10.3390/nu11092165, 2-s2.0-85072150683. PMC6769609 31509947

[66] Prananda A. T. , Dalimunthe A. , Harahap U. et al., *Phyllanthus emblica*: A Comprehensive Review of Its Phytochemical Composition and Pharmacological Properties, Frontiers in Pharmacology. (2023) 14, 10.3389/fphar.2023.1288618. PMC10637531 37954853

[67] Khaled S. E. , Hashem F. A.-M. , Shabana M. H. et al., A Biochemometric Approach for the Assessment of *Phyllanthus emblica* Female Fertility Effects as Determined via UPLC-ESI-qTOF-MS and GC-MS, Food & Function. (2019) 10, no. 8, 4620–4635, 10.1039/c9fo00767a, 2-s2.0-85070843231. 31290504

[68] Pandey K. B. and Rizvi S. I. , Plant Polyphenols as Dietary Antioxidants in Human Health and Disease, Oxidative Medicine and Cellular Longevity. (2009) 2, no. 5, 270–278, 10.4161/oxim.2.5.9498, 2-s2.0-77953474824. 20716914 PMC2835915

[69] Ekins S. , Mestres J. , and Testa B. , *In Silico* Pharmacology for Drug Discovery: Methods for Virtual Ligand Screening and Profiling, British Journal of Pharmacology. (2007) 152, no. 1, 9–20, 10.1038/sj.bjp.0707305, 2-s2.0-34548304745. 17549047 PMC1978274

[70] Li Y. , Han J. , Zhang Y. , Chen Y. , and Zhang Y. , Prophylactic Effect and Mechanism of p-Coumaric Acid Against Hypoxic Cerebral Edema in Mice, Respiratory Physiology & Neurobiology. (2019) 260, 95–104, 10.1016/j.resp.2018.11.004, 2-s2.0-85056694583. 30447305

[71] Rashno M. , Sarkaki A. , Farbood Y. et al., Possible Mechanisms Involved in the Neuroprotective Effects of Chrysin Against Mild Traumatic Brain Injury-Induced Spatial Cognitive Decline: An *In Vivo* Study in a Rat Model, Brain Research Bulletin. (2023) 204, 10.1016/j.brainresbull.2023. 37827266

[72] Kim Y. , Cho A. Y. , Kim H. C. , Ryu D. , Jo S. A. , and Jung Y. S. , Effects of Natural Polyphenols on Oxidative Stress-Mediated Blood-Brain Barrier Dysfunction, Antioxidants. (2022) 11, no. 2, 10.3390/antiox11020197. PMC8868362 35204080

[73] Horton J. D. , Goldstein J. L. , and Brown M. S. , SREBPs: Activators of the Complete Program of Cholesterol and Fatty Acid Synthesis in the Liver, The Journal of clinical investigation. (2002) 109, no. 9, 1125–1131, 10.1172/jci15593. 11994399 PMC150968

[74] Rosen E. D. , Walkey C. J. , Puigserver P. , and Spiegelman B. M. , Transcriptional Regulation of Adipogenesis, Genes & Development. (2000) 14, no. 11, 1293–1307, 10.1101/gad.14.11.1293. 10837022

[75] Shin M.-R. , Shin S. H. , and Roh S.-S. , *Diospyros kaki* and *Citrus unshiu* Mixture Improves Disorders of Lipid Metabolism in Nonalcoholic Fatty Liver Disease, Canadian Journal of Gastroenterology and Hepatology. (2020) 2020, 8812634–12, 10.1155/2020/8812634. 33425805 PMC7775147

[76] Murase T. , Misawa K. , Minegishi Y. et al., Coffee Polyphenols Suppress Diet-Induced Body Fat Accumulation by Downregulating SREBP-1c and Related Molecules in C57BL/6J Mice, American Journal of Physiology-Endocrinology and Metabolism. (2011) 300, no. 1, E122–E133, 10.1152/ajpendo.00441.2010, 2-s2.0-78650762536. 20943752

[77] Cuccioloni M. , Bonfili L. , Mozzicafreddo M. et al., A Ruthenium Derivative of Quercetin With Enhanced Cholesterol-Lowering Activity, RSC Advances. (2016) 6, no. 46, 39636–39641, 10.1039/c6ra06403e, 2-s2.0-84968756947.

[78] Matsusue K. , Aibara D. , Hayafuchi R. et al., Hepatic PPARγ and LXRα Independently Regulate Lipid Accumulation in the Livers of Genetically Obese Mice, FEBS Letters. (2014) 588, no. 14, 2277–2281, 10.1016/j.febslet.2014.05.012, 2-s2.0-84902659944. 24857376 PMC6301023

[79] Juvet L. K. , Andresen S. M. , Schuster G. U. et al., On the Role of Liver X Receptors in Lipid Accumulation in Adipocytes, Molecular Endocrinology. (2003) 17, no. 2, 172–182, 10.1210/me.2001-0210, 2-s2.0-0037312554. 12554745

[80] Lee S.-M. , Moon J. , Cho Y. , Chung J. H. , and Shin M.-J. , Quercetin Up-Regulates Expressions of Peroxisome Proliferator-Activated Receptor Γ, Liver X Receptor α, and ATP Binding Cassette Transporter A1 Genes and Increases Cholesterol Efflux in Human Macrophage Cell Line, Nutrition Research. (2013) 33, no. 2, 136–143, 10.1016/j.nutres.2012.11.010, 2-s2.0-84873525099. 23399664

[81] Ren K. , Jiang T. , and Zhao G.-J. , Quercetin Induces the Selective Uptake of HDL-Cholesterol via Promoting SR-BI Expression and the Activation of the PPARγ/LXRα Pathway, Food & Function. (2018) 9, no. 1, 624–635, 10.1039/c7fo01107e, 2-s2.0-85041196212. 29292466

[82] Chang Y.-C. , Lee T.-S. , and Chiang A.-N. , Quercetin Enhances ABCA1 Expression and Cholesterol Efflux Through a p38-Dependent Pathway in Macrophages, Journal of lipid research. (2012) 53, no. 9, 1840–1850, 10.1194/jlr.m024471, 2-s2.0-84864852278. 22711909 PMC3413225

[83] Li X.-Y. , Kong L.-X. , Li J. , He H.-X. , and Zhou Y.-D. , Kaempferol Suppresses Lipid Accumulation in Macrophages Through the Downregulation of Cluster of Differentiation 36 and the Upregulation of Scavenger Receptor Class B Type I and ATP-Binding Cassette Transporters A1 and G1, International Journal of Molecular Medicine. (2013) 31, no. 2, 331–338, 10.3892/ijmm.2012.1204, 2-s2.0-84873109054. 23232972

[84] Lu Y. and Jia Y.-P. , Quercetin Upregulates ABCA1 Expression Through Liver X Receptor Alpha Signaling Pathway in THP-1 Macrophages, European Review for Medical and Pharmacological Sciences. (2016) 20, no. 18, 3945–3952. 27735019

[85] Moon J. , Lee S. M. , Do H. J. , Cho Y. , Chung J. H. , and Shin M. J. , Quercetin Up‐Regulates LDL Receptor Expression in HepG2 Cells, Phytotherapy Research. (2012) 26, no. 11, 1688–1694, 10.1002/ptr.4646, 2-s2.0-84867863236. 22388943

[86] Ochiai A. , Miyata S. , Iwase M. , Shimizu M. , Inoue J. , and Sato R. , Kaempferol Stimulates Gene Expression of Low-Density Lipoprotein Receptor Through Activation of Sp1 in Cultured Hepatocytes, Scientific Reports. (2016) 6, no. 1, 1–10, 10.1038/srep24940, 2-s2.0-84964626294. 27109240 PMC4842988

[87] Baskaran G. , Salvamani S. , Ahmad S. A. , Shaharuddin N. A. , Pattiram P. D. , and Shukor M. Y. , HMG-CoA Reductase Inhibitory Activity and Phytocomponent Investigation of *Basella alba* Leaf Extract as a Treatment for Hypercholesterolemia, Drug Design, Development and Therapy. (2015) 9, 509–517, 10.2147/dddt.s75056, 2-s2.0-84921507513. 25609924 PMC4298350

[88] Dong S.-z. , Zhao S.-p. , Wu Z.-h. et al., Curcumin Promotes Cholesterol Efflux from Adipocytes Related to PPARgamma-LXRalpha-ABCA1 Passway, Molecular and Cellular Biochemistry. (2011) 358, no. 1-2, 281–285, 10.1007/s11010-011-0978-z, 2-s2.0-84879790955. 21748336

[89] Almatroodi S. A. , Alsahli M. A. , Almatroudi A. et al., Amla (*Emblica officinalis*): Role in Health Management via Controlling Various Biological Activities, Gene Reports. (2020) 21, 10.1016/j.genrep.2020.100820.

[90] Escorcia W. , Ruter D. L. , Nhan J. , and Curran S. P. , Quantification of Lipid Abundance and Evaluation of Lipid Distribution in *Caenorhabditis elegans* by Nile Red and Oil Red O Staining, Journal of Visualized Experiments. (2018) 133, 10.3791/57352, 2-s2.0-85044791298. PMC5931440 29553519

[91] Mopuri R. , Kalyesubula M. , Rosov A. , Edery N. , Moallem U. , and Dvir H. , Improved Folch Method for Liver-Fat Quantification, Frontiers in Veterinary Science. (2021) 7, 10.3389/fvets.2020.594853. PMC7835396 33511163

[92] Sato R. , Buesa L. M. , and Nerurkar P. V. , Anti‐Obesity Effects of *Emblica officinalis* (Amla) Are Associated With Inhibition of Nuclear Transcription Factor, Peroxisome Proliferator‐Activated Receptor Gamma (PPARγ), The FASEB Journal. (2010) 24, no. S1, 661–664, 10.1096/fasebj.24.1_supplement.661.4.

