# Supplementary material for: The Constituents of Phyllanthus emblica Fruit Ameliorate Hyperlipidemia Through the Modulation of SREBPs, HMG‐CoA Reductase, and LDL Receptor Pathway
Source: Scientifica (Cairo). 2025 Dec 30;2025:7941857. doi: 10.1155/sci5/7941857 (PMC12782344; doi:10.1155/sci5/7941857)
Supplement: Supplementary file 1 — Supporting Information Additional supporting information can be found online in the Supporting Information section. [file SCI5-2025-7941857-s001.docx]

**Supplementary Information**

**SUPPLEMENTARY TABLE 1**: Pharmacological activities of several compounds present in the *Phyllanthus emblica* fruit pulp

| **No.** | **Compound** | **Pharmacologic activity** | **Protein involved** | **Reference** |
| --- | --- | --- | --- | --- |
| 1 | Gallic acid | Ameliorates dyslipidemia, hepatosteatosis, and oxidative stress in rats. cardioprotective, and anti-inflammatory | SREBP-1c, ACC, HMGCR, PPARα, and FAS | (Doan et al., 2015; Lee et al., 2021) |
| 2 | Ellagic acid | Reduces the production of TG and cholesterol, stimulates lipid metabolism | FABP4, PPARγ, C/EBPα | (Aranaz et al., 2019; Park et al., 2011) |
| 3 | Quercetin | Anti-inflammatory, antioxidant, and reduce cholesterol and fatty acid synthesis | SREBP-2, ACC, HMGCR, PPARγ, and FABP4 | (Damiano, Giannotti, Gnoni, Siculella, & Gnoni, 2019; Sahebkar, 2017) |
| 4 | Betaine | Suppresses accumulation of white adipose, TG, TC, and LDL cholesterol | PPARα, PPARγ, SREBP-1c, SCD-1, DGAT, E/EBPα | (Du et al., 2018; D. H. Kim, Sung, Chung, & Kim, 2014) |
| 5 | Trigonelline | Inhibit the expression of genes involved in lipid synthesis, increase cholesterol efflux | FAS, ACC, PPARα, PPARγ, ABCA1 | (Sharma, Lone, Knott, Hassan, & Abdullah, 2018) |
| 6 | Myricitrin | Antioxidant, Free radical scavenger, Lipid peroxidase inhibitor, Kinase inhibitor, Lactase inhibitor, Sugar-phosphatase inhibitor | NRF-2, TNFα, Caspase-3, Caspase-9, ERK, HO-1, NF-κB | (Gao, Liu, Zhang, Sun, & Wang, 2019; B. Zhang et al., 2017) |
| 7 | Myricetin | Myricetin ameliorates atherosclerosis and reduces LDL cholesterol, glucose, and TG. | MCP-1, IL-6, PPARα, LDLR, PPARγ, E/EBPα | (Meng, Wang, Xing, Liu, & Li, 2019; Su, Feng, Zheng, & Chen, 2016) |
| 8 | Leucine | Anti-obesity effects on glucose tolerance, lipid metabolism, and insulin sensitivity. | AMPK, UCP-1, PPARγ, FABP1, TNFα, | (Torres-Leal et al., 2011; L. Zhang et al., 2020) |
| 9 | Kaempferol | Reduce cholesterol synthesis, increase uptake of LDL cholesterol from the bloodstream | SREBP-2, SREBP-1c, HMGCR, LDLR, AMPK | (Eilam et al., 2022; Ochiai et al., 2016) |
| 10 | Paromomycin | Protein synthesis inhibitor, antibacterial, inhibits phospholipid synthesis | Lactase dehydrogenase (LDH), Aminoglycoside phosphotransferase (APH), | (Mehta & Champney, 2003) |
| 11 | Rutin | Antioxidant, antidiabetic, anti-inflammatory, neuroprotective, nephroprotective, and hepatoprotective effects | GLUT4, AMPK, PEPCK, TNFα, | (Ghorbani, 2017; Yang, Guo, & Yuan, 2008) |
| 12 | Caffeic acid | Modify adipocyte function, differentiation, and lipid metabolism. | C/EBPα, ERK1/2, PPARγ, FABP4, SREBP-1c | (Vasileva et al., 2020; Veeren et al., 2021) |
| 13 | Quinic acid | Anti-adipogenic, lipolytic, antioxidant, anti-bacterial | PKC, MAPK, LXRα, FAS, | (Benali et al., 2022; Wu et al., 2015) |
| 14 | Catechin | Increase cholesterol efflux and reduce the accumulation of cholesterol in cells. | PPARα, SREBP-1c, FABP4, NF-κB, PPARγ | (Hursel & Westerterp-Plantenga, 2013; A. Kim et al., 2011) |
| 15 | Chrysin | Inhibits the production of lipids and cholesterol in the liver. It also controls the expression of several genes involved in lipid metabolism | AMPK, SREBP-2, SREBP-1c, TNFα, UCP1, PPARα | (Jana et al., 2008; Oriquat et al., 2023) |
| 16 | Methyl gallate | Antioxidant, inhibit the differentiation of preadipocytes to mature adipocyte, induces autophagy and lysosome formation | ACC, FAS, PPARα, ABCA1, | (Rummun et al., 2021; Whang et al., 2005) |
| 17 | Ellagitannin | Antioxidant, anti-inflammatory, reduces body weight and cholesterol | PPARα, AMPK, α- and β-Galactosidase | (Fotschki et al., 2018; Żary-Sikorska et al., 2020) |
| 18 | Malic acid | Increases fatty acid oxidation and lipid metabolism. | FAS, ACC, PPARγ | (Raybaudi-Massilia, Mosqueda-Melgar, & Martín-Belloso, 2009) |
| 19 | Pedunculagin | Antioxidant, Anti-inflammatory, Hepatic disorder treatment, Free radical scavenger | HMGCR, PPARγ, SREBP2, ACC, | (Silva Fernandes et al., 2022) |
| 20 | Coumaric acid | Modulates glucose and lipid metabolism.  Ameliorate Obesity. | AMPK, ACC, CPT-1, PPARα and 2-NBDG uptake. PPARγ, C/EBPα, Leptin and Adiponectin | (Hsu, Wu, Huang, & Yen, 2009; Yoon et al., 2013) |
| 21 | Betulinic acid | Anti-obesity effect | PPARγ, C/EBPα, PI3K and AKT | (Savova et al., 2021) |

**SUPPLEMENTARY TABLE 2:** Composition of the normal diet (Control), the high-fat diet (HFD), normal diet supplemented with 2% (w/w) dried power of P. emblica fruit (Control + PEF), and high-fat diet supplemented with 2% (w/w) dried power of P. emblica fruit (HFD + PEF)

| **Ingredient** | **Normal diet (Control)** | **Control + PEF** | **High-fat diet (HFD)** | **HFD + PEF** |
| --- | --- | --- | --- | --- |
|  | **(g/kg diet)** | **(g/kg diet)** | **(g/kg diet)** | **(g/kg diet)** |
| Corn starch | 600 | 587 | 400 | 387 |
| PEF | 0 | 20 | 0 | 20 |
| Casein | 230 | 230 | 210 | 210 |
| Corn oil | 50 | 50 | 0 | 0 |
| Cellulose | 70 | 63 | 40 | 33 |
| Beef tallow | 0 | 0 | 300 | 300 |
| Vitamin mix | 10 | 10 | 10 | 10 |
| Mineral mix | 30 | 30 | 30 | 30 |
| Methionine | 3 | 3 | 3 | 3 |
| Choline | 2 | 2 | 2 | 2 |
| Salt | 5 | 5 | 5 | 5 |
| Total weight (g) | 1000 | 1000 | 1000 | 1000 |
| kcal/kg diet | 3782 | 3783 | 5152 | 5153 |
| Calorie from fat (%) | 11.9 | 11.9 | 52.4 | 52.4 |

The energy content of each of the 100 g ingredients are as follows: corn starch 400 Kcal, PEF 266 kcal, casein 400 kcal, corn oil 900 kcal, cellulose 0 kcal, beef tallow 900 kcal, and methionine 400 kcal. Other ingredient such as vitamin mix, mineral mix, choline, and salt contains no energy (Parvez, Jashin, Yesmin, Reza, & Akter, 2020).

**SUPPLEMENTARY TABLE 3**: Cytochrome enzyme inhibitory activities, leadlikenss, and synthetic accessibility of selected compounds of *P. emblica* fruit predicted by SWISS prediction tool

|  | **Molecule name** | **CYP1A2 inhibitor** | **CYP2C19  inhibitor** | **CYP2C9 inhibitor** | **CYP2D6  inhibitor** | **CYP3A4  inhibitor** | **Leadlikeness (#violations)** | **Synthetic Accessibility** |
| --- | --- | --- | --- | --- | --- | --- | --- | --- |
| 1 | Gallic acid | No | No | No | No | Yes | 1 | 1.22 |
| 2 | Ellagic acid | Yes | No | No | No | No | 0 | 3.17 |
| 3 | Quercetin | Yes | No | No | Yes | Yes | 0 | 3.23 |
| 4 | Betaine | No | No | No | No | No | 1 | 1.0 |
| 5 | Trigonelline | No | No | No | No | No | 1 | 1.04 |
| 6 | Myricitrin | No | No | No | No | No | 1 | 5.32 |
| 7 | Myricetin | Yes | No | No | No | Yes | 0 | 3.27 |
| 8 | Leucine | No | No | No | No | No | 1 | 1.39 |
| 9 | Kaempferol | Yes | No | No | Yes | Yes | 0 | 3.14 |
| 10 | Paromomycin | No | No | No | No | No | 2 | 7.37 |
| 11 | Rutin | No | No | No | No | No | 1 | 6.52 |
| 12 | Caffeic acid | No | No | No | No | No | 1 | 1.81 |
| 13 | Quinic acid | No | No | No | No | No | 1 | 3.34 |
| 14 | Catechin | No | No | No | No | No | 0 | 3.5 |
| 15 | Chrysin | Yes | No | No | Yes | Yes | 1 | 2.93 |
| 16 | Methyl gallate | No | No | No | No | No | 1 | 1.5 |
| 17 | Ellagitannin | No | No | No | No | No | 1 | 8.33 |
| 18 | Malic acid | No | No | No | No | No | 1 | 2.27 |
| 19 | Pedunculagin | No | No | No | No | No | 1 | 6.68 |
| 20 | Coumaric acid | Yes | No | No | No | No | 2 | 5.26 |
| 21 | Betulinic acid | No | No | No | No | No | 3 | 6.02 |
| 22 | Atorvastatin | No | Yes | No | Yes | Yes | 3 | 4.95 |


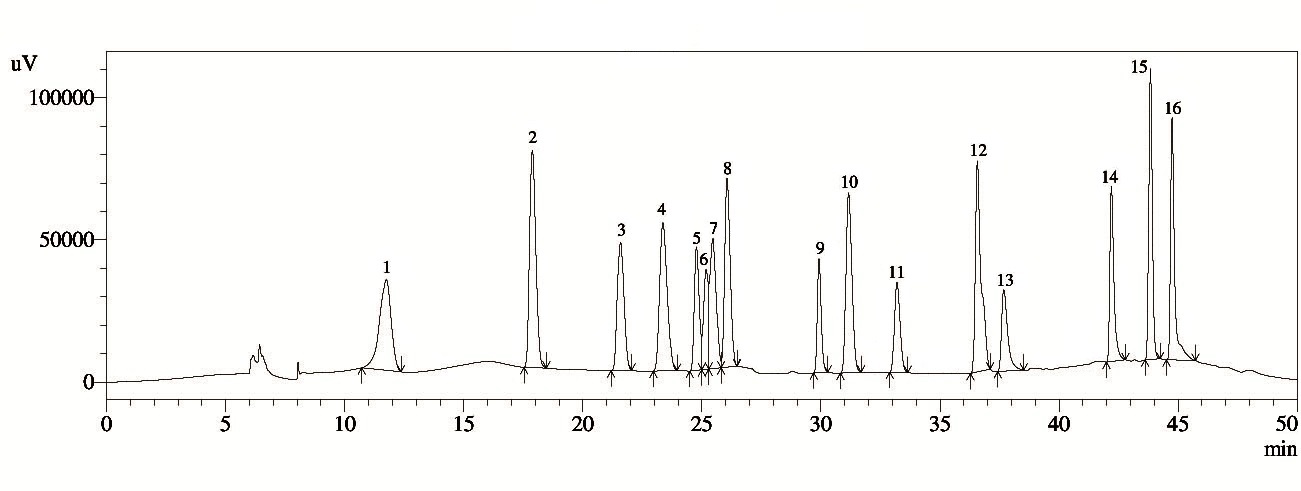


**SUPPLEMENTARY FIGURE 1:** 1. gallic acid, 2. quinic acid, 3. leucine, 4. malic acid, 5. catechin, 6. coumaric acid, 7. betaine, 8. Trigonelline, 9. ellagic acid, 10. caffeic acid, 11. chrysin, 12. myricetin, 13. methyl gallate, 14. kaempferol, 15. quercetin, and 16. betulinic acid.

**Method of lipid extraction and absorbance measurement:** Lipid was extracted from the hepatic tissue of rats according to a modified Folch extraction method (Mopuri et al., 2021). Briefly, 1 g (equivalent to 1 ml) of hepatic tissue was homogenized thoroughly in a Potter-Elvehjem tissue homogenizer with a 25 ml mixture of methanol and chloroform (1:2). The mixture was sonicated for 5 min, followed by centrifugation at 3000 g. The supernatant was discarded, and the lower part containing the lipids was carefully collected and washed with 0.9% NaCl solution (200 µl for each ml residue) to remove protein, peptide, DNA, RNA, and other hydrophilic contaminants. The purified lipid-rich portion was evaporated in a fume hood at room temperature to remove the residual chloroform and moisture. The lipid residue was dissolved in isopropanol, and volume was adjusted to 1 ml. The resulting lipid solution was mixed with freshly prepared Oil Red O (ORO) working solution (0.5%), followed by incubation at room temperature for 30 minutes to remove undissolved particles before imaging. For visual comparison, 2 ml of solution from each sample was placed in a 30 mm x 15 mm Petri dish. Photograph of the petri dishes from all four groups of rats was taken using a Nikon D7500 (20.9-megapixel) digital camera under identical exposure settings, distance, and lighting (Ramírez-Zacarías et al., 1992). Additionally, the samples were diluted 40-fold with a solution of ethanol and water (2:3), and absorbance values were recorded at 518 nm using a spectrophotometer (Escorcia et al., 2018).

**Results:** Oil Red O (ORO) staining demonstrates that HFD consumption markedly increased the lipid accumulation in the liver compared with the control group. PEF could not alter lipid accumulation in either the control or HFD-consuming rats. Similarly, the absorbance was significantly increased due to the consumption of HFD. However, the PEF-supplemented diet was unable to change the lipid accumulation in both the control diet and HFD-consuming groups, indicating the inability of PEF to reduce the hepatic lipid accumulation.


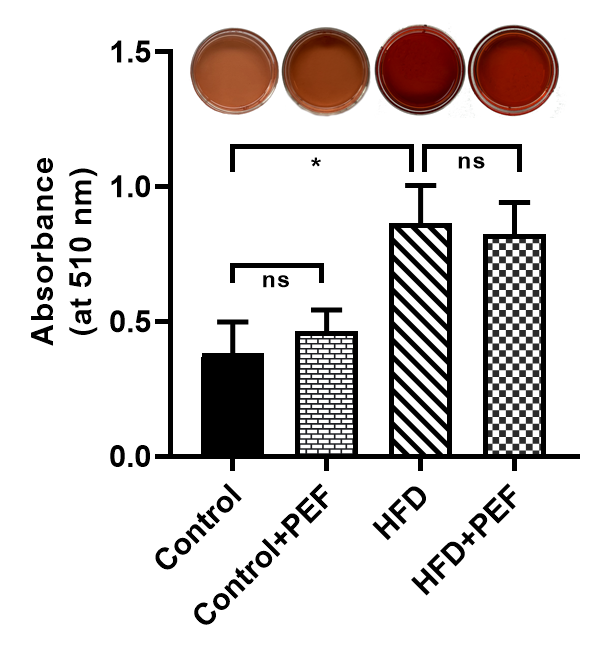


**SUPPLEMENTARY FIGURE 2:** Change in hepatic lipid accumulation due to *P. emblica* (PEF) supplementation with control diet and high-fat diet (HFD). The rats were provided with any one of the following four diets: normal diet (Control), control diet containing 2% (w/w) dried powder of *P. emblica* fruit (Control + PEF), HFD, or HFD containing 2% PEF (HFD + PEF). After 8 weeks of feeding, the rats were sacrificed, liver samples were collected, and lipid was extracted from 1 g of hepatic tissue of each rat. The lipid was reconstituted in isopropanol to make a 1 ml solution, which was mixed with a working solution of Oil Red O (ORO). This 2 ml solution from each sample was place in a 30 mm x 15 mm Petri dish for visual comparison. Photograph of the Petri dish was captured under identical exposure settings, distance, and lighting (upper). Additionally, the samples were diluted 40-fold with a solution of ethanol and water (2:3), and absorbance values were recorded at 518 nm (lower). Values are presented as mean ± SEM, n = 6. For comparison, One-way ANOVA was conducted, followed by Newman-Keuls multiple comparison tests. Mean values are considered significantly different when p < 0.05, which is represented by an asterisk (*).

**Supplementary Abbreviations:**

ABCA1: ATP-binding cassette subfamily A member 1

SREBP-1: Sterol regulatory element-binding protein 1

PPARγ: Peroxisome proliferator-activated receptor gamma

C/EBPα: CCAAT/enhancer-binding protein alpha

PI3K: Phosphoinositide 3-kinase

AKT: Protein kinase B

AMPK: AMP-activated protein kinase

ACC: Acetyl-CoA carboxylase

CPT-1: Carnitine palmitoyl transferase I

2-NBDG: 2-[N-(7-nitrobenz-2-oxa-1,3-diazol-4-yl) amino]-2-deoxy-D-glucose

TRPV1: Transient receptor potential vanilloid subtype 1

TRPM8: transient receptor potential melastatin 8

PKC: Protein kinase C

PKA: Protein Kinase A

Mcp-1: Monocyte chemoattractant protein-1

TXNIP: Thioredoxin interacting protein

GLUT1: Glucose transporter 1

FAS: Fatty acid synthase

GPAT: Glycerol phosphate acyltransferase

HMGCR: Hydroxymethylglutaryl-coenzyme A reductase

TNFα: Tumor necrosis factor α

UCP1: Mitochondrial uncoupling protein 1

FABP4: Fatty acid binding protein 4

NF-κB: Nuclear factor kappa B

MAPK: Mitogen-activated protein kinase

LXRα: Liver X receptor

ERK1/2: Extracellular signal-regulated kinase

SCD-1: Stearoyl CoA desaturase 1

DGAT: Diglyceride acyltransferase

E/EBPα: CCAAT enhancer binding protein alpha

AST: Aspartate transaminase

ALT: Alanine transaminase

SOD: Superoxide dismutase

GLUT4: Glucose transporter 4

PEPCK: Phosphoenolpyruvate carboxykinase

IL-6: Interleukin 6

NRF-2: Nuclear factor erythroid 2-related factor 2

ERK: Extracellular signal-regulated kinase

HO-1: Heme oxygenase 1

**Supplementary References:**

Aranaz, P., Navarro-Herrera, D., Zabala, M., Miguéliz, I., Romo-Hualde, A., López-Yoldi, M., Martínez, J. A., Vizmanos, J. L., Milagro, F. I. & González-Navarro, C. J. (2019). Phenolic compounds inhibit 3T3-L1 adipogenesis depending on the stage of differentiation and their binding affinity to PPARγ. *Molecules, 24*(6), 1045.

Benali, T., Bakrim, S., Ghchime, R., Benkhaira, N., El Omari, N., Balahbib, A., Taha, D., Zengin, G., Hasan, M. M., Bibi, S. & Bouyahya, A. (2022). Pharmacological insights into the multifaceted biological properties of quinic acid. *Biotechnology and Genetic Engineering Reviews*, 1-30.

Damiano, F., Giannotti, L., Gnoni, G. V., Siculella, L., & Gnoni, A. (2019). Quercetin inhibition of SREBPs and ChREBP expression results in reduced cholesterol and fatty acid synthesis in C6 glioma cells. *The international journal of biochemistry & cell biology, 117*, 105618.

Doan, K. V., Ko, C. M., Kinyua, A. W., Yang, D. J., Choi, Y. H., Oh, I. Y., Nguyen, N. M., Ko, A., Choi, J. W., Jeong, Y. & Jung, M. H. (2015). Gallic acid regulates body weight and glucose homeostasis through AMPK activation. *Endocrinology, 156*(1), 157-168.

Du, J., Shen, L., Tan, Z., Zhang, P., Zhao, X., Xu, Y., Gan, M., Yang, Q., Ma, J., Jiang, A. A. & Tang, G. (2018). Betaine supplementation enhances lipid metabolism and improves insulin resistance in mice fed a high-fat diet. *Nutrients, 10*(2), 131.

Eilam, Y., Pintel, N., Khattib, H., Shagug, N., Taha, R., & Avni, D. (2022). Regulation of cholesterol metabolism by phytochemicals derived from algae and edible mushrooms in non-alcoholic fatty liver disease. *International Journal of Molecular Sciences, 23*(22), 13667.

Escorcia, W., Ruter, D. L., Nhan, J., & Curran, S. P. (2018). Quantification of lipid abundance and evaluation of lipid distribution in Caenorhabditis elegans by Nile Red and Oil Red O staining. Journal of Visualized Experiments, 133, 57352. https://doi.org/10.3791/57352.

Fotschki, B., Juśkiewicz, J., Kołodziejczyk, K., Jurgoński, A., Kosmala, M., Milala, J., Ognik, K. Zduńczyk, Z. (2018). Protective effects of ellagitannin-rich strawberry extracts on biochemical and metabolic disturbances in rats fed a diet high in fructose. *Nutrients, 10*(4), 445.

Gao, J., Liu, C., Zhang, H., Sun, Z., & Wang, R. (2019). Myricitrin exhibits anti-atherosclerotic and anti-hyperlipidemic effects in diet-induced hypercholesterolemic rats. *AMB Express, 9*(1), 1-7.

Ghorbani, A. (2017). Mechanisms of antidiabetic effects of flavonoid rutin. *Biomedicine & Pharmacotherapy, 96*, 305-312.

Hsu, C.-L., Wu, C.-H., Huang, S.-L., & Yen, G.-C. (2009). Phenolic compounds rutin and o-coumaric acid ameliorate obesity induced by high-fat diet in rats. *Journal of agricultural and food chemistry, 57*(2), 425-431.

Hursel, R., & Westerterp-Plantenga, M. S. (2013). Catechin-and caffeine-rich teas for control of body weight in humans. *The American journal of clinical nutrition, 98*(6), 1682S-1693S.

Jana, K., Yin, X., Schiffer, R. B., Chen, J. J., Pandey, A. K., Stocco, D. M., Grammas, P. & Wang, X.(2008). Chrysin, a natural flavonoid enhances steroidogenesis and steroidogenic acute regulatory protein gene expression in mouse Leydig cells. *Journal of Endocrinology, 197*(2), 315-324.

Kim, A., Chiu, A., Barone, M. K., Avino, D., Wang, F., Coleman, C. I., & Phung, O. J. (2011). Green tea catechins decrease total and low-density lipoprotein cholesterol: a systematic review and meta-analysis. *Journal of the American Dietetic Association, 111*(11), 1720-1729.

Kim, D. H., Sung, B., Chung, H. Y., & Kim, N. D. (2014). Modulation of colitis-associated colon tumorigenesis by baicalein and betaine. *Journal of Cancer Prevention, 19*(3), 153.

Lee, A.-T., Yang, M.-Y., Lee, Y.-J., Yang, T.-W., Wang, C.-C., & Wang, C.-J. (2021). Gallic acid improves diabetic steatosis by downregulating microRNA-34a-5p through targeting NFE2L2 expression in high-fat diet-fed db/db mice. *Antioxidants, 11*(1), 92.

Mehta, R., & Champney, W. S. (2003). Neomycin and paromomycin inhibit 30S ribosomal subunit assembly in Staphylococcus aureus. *Current microbiology, 47*, 0237-0243.

Meng, Z., Wang, M., Xing, J., Liu, Y., & Li, H. (2019). Myricetin ameliorates atherosclerosis in the low-density-lipoprotein receptor knockout mice by suppression of cholesterol accumulation in macrophage foam cells. *Nutrition & metabolism, 16*(1), 1-9.

Mopuri, R., Kalyesubula, M., Rosov, A., Edery, N., Moallem, U., & Dvir, H. (2021). Improved Folch method for liver-fat quantification. Frontiers in Veterinary Science, 7, 594853. https://doi.org/10.3389/fvets.2020.594853.

Ochiai, A., Miyata, S., Iwase, M., Shimizu, M., Inoue, J., & Sato, R. (2016). Kaempferol stimulates gene expression of low-density lipoprotein receptor through activation of Sp1 in cultured hepatocytes. *Scientific Reports, 6*(1), 24940.

Oriquat, G., Masoud, I. M., Kamel, M. A., Aboudeya, H. M., Bakir, M. B., & Shaker, S. A. (2023). The Anti-Obesity and Anti-Steatotic Effects of Chrysin in a Rat Model of Obesity Mediated through Modulating the Hepatic AMPK/mTOR/lipogenesis Pathways. *Molecules, 28*(4), 1734.

Park, S.-H., Kim, J.-L., Lee, E.-S., Han, S.-Y., Gong, J.-H., Kang, M.-K., & Kang, Y.-H. (2011). Dietary ellagic acid attenuates oxidized LDL uptake and stimulates cholesterol efflux in murine macrophages. *The Journal of nutrition, 141*(11), 1931-1937.

Parvez, M., Jashin, N., Yesmin, M., Reza, M., & Akter, N. (2020). Proximate, phytochemical and antioxidant activity of amla powder and amla candy. Journal of Environmental Science and Natural Resources, 13(1-2), 82-86.

Ramírez-Zacarías, J. L., Castro-Muñozledo, F., & Kuri-Harcuch, W. (1992). Quantitation of adipose conversion and triglycerides by staining intracytoplasmic lipids with Oil Red O. Histochemistry, 97(6), 493–497. https://doi.org/10.1007/BF00316069.

Raybaudi-Massilia, R. M., Mosqueda-Melgar, J., & Martín-Belloso, O. (2009). Antimicrobial activity of malic acid against Listeria monocytogenes, Salmonella Enteritidis and Escherichia coli O157: H7 in apple, pear and melon juices. *Food Control, 20*(2), 105-112.

Rummun, N., Pires, E., McCullagh, J., Claridge, T. W., Bahorun, T., Li, W.-W., & Neergheen, V. S. (2021). Methyl gallate–Rich fraction of Syzygium coriaceum leaf extract induced cancer cell cytotoxicity via oxidative stress. *South African Journal of Botany, 137*, 149-158.

Sahebkar, A. (2017). Effects of quercetin supplementation on lipid profile: A systematic review and meta-analysis of randomized controlled trials. *Critical reviews in food science and nutrition, 57*(4), 666-676.

Savova, M. S., Vasileva, L. V., Mladenova, S. G., Amirova, K. M., Ferrante, C., Orlando, G., Wabitsch, M. & Georgiev, M. I. (2021). Ziziphus jujuba Mill. leaf extract restrains adipogenesis by targeting PI3K/AKT signaling pathway. *Biomedicine & Pharmacotherapy, 141*, 111934.

Sharma, L., Lone, N. A., Knott, R. M., Hassan, A., & Abdullah, T. (2018). Trigonelline prevents high cholesterol and high fat diet induced hepatic lipid accumulation and lipo-toxicity in C57BL/6J mice, via restoration of hepatic autophagy. *Food and chemical toxicology, 121*, 283-296.

Silva Fernandes, A., Hollanda Véras, J., Silva, L. S., Puga, S. C., Luiz Cardoso Bailão, E. F., de Oliveira, M. G., Cardoso, C. G., Carneiro, C. C., Costa Santos, S. D. & Chen-Chen, L. (2022). Pedunculagin isolated from Plinia cauliflora seeds exhibits genotoxic, antigenotoxic and cytotoxic effects in bacteria and human lymphocytes. *Journal of Toxicology and Environmental Health, Part A, 85*(9), 353-363.

Su, H.-m., Feng, L.-n., Zheng, X.-d., & Chen, W. (2016). Myricetin protects against diet-induced obesity and ameliorates oxidative stress in C57BL/6 mice. *Journal of Zhejiang University. Science. B, 17*(6), 437.

Torres-Leal, F. L., Fonseca-Alaniz, M. H., Teodoro, G. F., de Capitani, M. D., Vianna, D., Pantaleão, L. C., Matos-Neto, E. M., Rogero, M. M., Donato, J. & Tirapegui, J. (2011). Leucine supplementation improves adiponectin and total cholesterol concentrations despite the lack of changes in adiposity or glucose homeostasis in rats previously exposed to a high-fat diet. *Nutrition & metabolism, 8*(1), 1-10.

Vasileva, L. V., Savova, M. S., Amirova, K. M., Balcheva-Sivenova, Z., Ferrante, C., Orlando, G., Wabitsch, M. & Georgiev, M. I. (2020). Caffeic and chlorogenic acids synergistically activate browning program in human adipocytes: Implications of AMPK-and PPAR-mediated pathways. *International Journal of Molecular Sciences, 21*(24), 9740.

Veeren, B., Bringart, M., Turpin, C., Rondeau, P., Planesse, C., Ait-Arsa, I., Gimié, F., Marodon, C., Meilhac, O., Gonthier, M. P. & Diotel, N. (2021). Caffeic Acid, One of the Major Phenolic Acids of the Medicinal Plant Antirhea borbonica, Reduces Renal Tubulointerstitial Fibrosis. *Biomedicines, 9*(4), 358.

Whang, W. K., Park, H. S., Ham, I., Oh, M., Namkoong, H., Kim, H. K., Hwang, D. W., Hur, S. Y., Kim, T. E., Park, Y. G. & Kim, J. R. (2005). Methyl gallate and chemicals structurally related to methyl gallate protect human umbilical vein endothelial cells from oxidative stress. *Experimental & Molecular Medicine, 37*(4), 343-352.

Wu, C., Luan, H., Wang, S., Zhang, X., Liu, H., & Guo, P. (2015). Pandanus tectorius derived caffeoylquinic acids inhibit lipid accumulation in HepG2 hepatoma cells through regulation of gene expression involved in lipid metabolism. *Yao xue xue bao= Acta Pharmaceutica Sinica, 50*(3), 278-283.

Yang, J., Guo, J., & Yuan, J. (2008). In vitro antioxidant properties of rutin. *LWT-Food Science and Technology, 41*(6), 1060-1066.

Yoon, S.-A., Kang, S.-I., Shin, H.-S., Kang, S.-W., Kim, J.-H., Ko, H.-C., & Kim, S.-J. (2013). p-Coumaric acid modulates glucose and lipid metabolism via AMP-activated protein kinase in L6 skeletal muscle cells. *Biochemical and biophysical research communications, 432*(4), 553-557.

Żary-Sikorska, E., Fotschki, B., Jurgoński, A., Kosmala, M., Milala, J., Kołodziejczyk, K., Majewski, M., Ognik, K. & Juśkiewicz, J. (2020). Protective effects of a strawberry ellagitannin-rich extract against pro-oxidative and pro-inflammatory dysfunctions induced by a high-fat diet in a rat model. *Molecules, 25*(24), 5874.

Zhang, B., Shen, Q., Chen, Y., Pan, R., Kuang, S., Liu, G., Sun, G. & Sun, X. (2017). Myricitrin alleviates oxidative stress-induced inflammation and apoptosis and protects mice against diabetic cardiomyopathy. *Scientific Reports, 7*(1), 44239.

Zhang, L., Li, F., Guo, Q., Duan, Y., Wang, W., Zhong, Y., Yang, Y. & Yin, Y. (2020). Leucine supplementation: a novel strategy for modulating lipid metabolism and energy homeostasis. *Nutrients, 12*(5), 1299.
